# A multi-organoid platform identifies CIART as a key factor for SARS-CoV-2 infection

**DOI:** 10.1038/s41556-023-01095-y

**Published:** 2023-03-13

**Authors:** Xuming Tang, Dongxiang Xue, Tuo Zhang, Benjamin E. Nilsson-Payant, Lucia Carrau, Xiaohua Duan, Miriam Gordillo, Adrian Y. Tan, Yunping Qiu, Jenny Xiang, Robert E. Schwartz, Benjamin R. tenOever, Todd Evans, Shuibing Chen

**Affiliations:** 1 Department of Surgery, Weill Cornell Medicine, New York, NY USA; 2 Center for Genomic Health, Weill Cornell Medicine, New York, NY USA; 3 Genomics Resources Core Facility, Weill Cornell Medicine, New York, NY USA; 4 Department of Microbiology, New York University, New York, NY USA; 5 Stable Isotope and Metabolomics Core Facility, The Einstein-Mount Sinai Diabetes Research Center, Albert Einstein College of Medicine, Bronx, New York USA; 6 Division of Gastroenterology and Hepatology, Department of Medicine, Weill Cornell Medicine, New York, NY USA; 7 Department of Physiology, Biophysics and Systems Biology, Weill Cornell Medicine, New York, NY USA; 8 Present Address: TWINCORE Centre for Experimental and Clinical Infection Research, Hannover, Germany

**Keywords:** Embryonic stem cells, SARS-CoV-2

## Abstract

COVID-19 is a systemic disease involving multiple organs. We previously established a platform to derive organoids and cells from human pluripotent stem cells to model SARS-CoV-2 infection and perform drug screens^1,2^. This provided insight into cellular tropism and the host response, yet the molecular mechanisms regulating SARS-CoV-2 infection remain poorly defined. Here we systematically examined changes in transcript profiles caused by SARS-CoV-2 infection at different multiplicities of infection for lung airway organoids, lung alveolar organoids and cardiomyocytes, and identified several genes that are generally implicated in controlling SARS-CoV-2 infection, including *CIART*, the circadian-associated repressor of transcription. Lung airway organoids, lung alveolar organoids and cardiomyocytes derived from isogenic *CIART*^−/−^ human pluripotent stem cells were significantly resistant to SARS-CoV-2 infection, independently of viral entry. Single-cell RNA-sequencing analysis further validated the decreased levels of SARS-CoV-2 infection in ciliated-like cells of lung airway organoids. CUT&RUN, ATAC-seq and RNA-sequencing analyses showed that *CIART* controls SARS-CoV-2 infection at least in part through the regulation of *NR4A1*, a gene also identified from the multi-organoid analysis. Finally, transcriptional profiling and pharmacological inhibition led to the discovery that the Retinoid X Receptor pathway regulates SARS-CoV-2 infection downstream of CIART and NR4A1. The multi-organoid platform identified the role of circadian-clock regulation in SARS-CoV-2 infection, which provides potential therapeutic targets for protection against COVID-19 across organ systems.

## Abstract (web summary)

Systematic infection of a human multi-organoid system shows that deficiency in the host factor CIART impairs SARS-CoV-2 infection through downregulation of the RXR pathway and subsequent impairment of fatty-acid synthesis.

## Main

Coronavirus disease 19 (COVID-19) is a multi-organ disease caused by infection of severe acute respiratory syndrome coronavirus 2 (SARS-CoV-2). Although SARS-CoV-2 primarily infects the respiratory tract, patients with COVID-19 present with a wide range of disease indications, including the gastrointestinal, cardiovascular and neurological systems. Organoid models, derived from human pluripotent stem cells (hPSCs) or adult tissues, have proven to be powerful tools to study viral tropism and the host response, and have also been used for drug screens. A wide range of human cells are permissive for SARS-CoV-2 infection, including those in lung alveolar^2–7^, lung airway^5,8^, small intestine and colon^2^, brain^9–12^, choroid plexus^13^, heart^1,14–21^, liver, pancreas^22^, kidney^23^, blood vessels^24^ and tonsil^25^ organoids. Although insight into cellular tropism and host responses has been gained, certain key aspects underlying the regulation of infection remain undetermined. In particular, it is unknown whether common factors could be targeted to block or resist SARS-CoV-2 infection across distinct tissue types, which would be a great advantage for the treatment of multi-systemic manifestations of the disease. To address this issue, we performed a systematic analysis of transcriptional changes caused by SARS-CoV-2 infection across several distinct hPSC-derived cell types and organoids. We identified *CIART*, a nuclear transcription factor, as a key regulator of SARS-CoV-2 infection. *CIART* (also known as *CHRONO*, *C1orf51* and *GM129*) was originally identified as a regulator of a circadian-clock feedback loop^26–28^. Although *CIART* has not been associated with human disease, how the circadian rhythm of host cells may impact viral replication is an area of emerging interest. A recent study using the Calu-3 lung cancer cell line suggests that knockdown of *Bmal1* inhibits SARS-CoV-2 entry^29^. Here we systematically analysed the biological role and downstream mechanism of *CIART* regulation in SARS-CoV-2 infection.

**Fig. 1:**
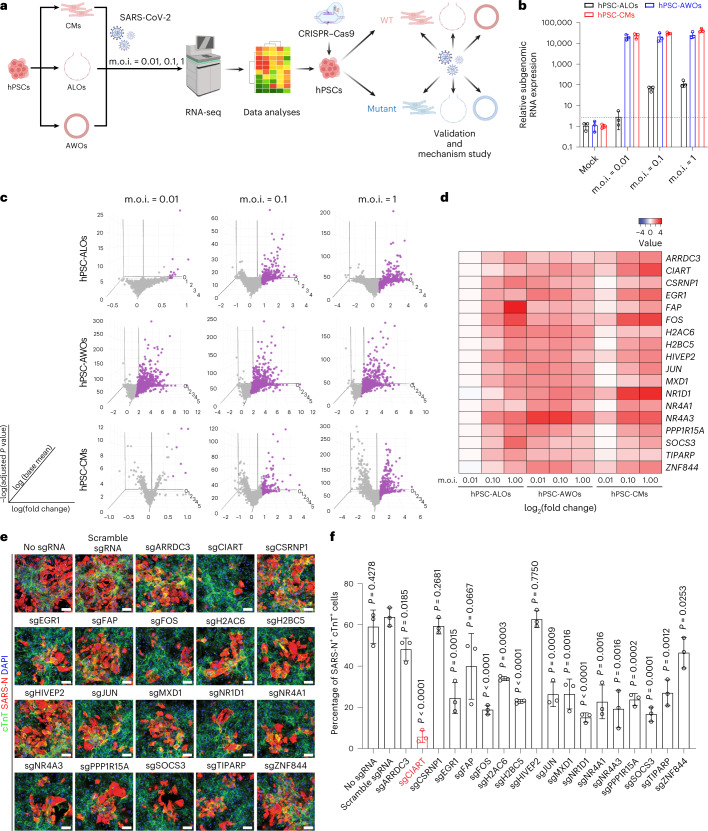
A multi-organoid platform to identify genes involved in SARS-CoV-2 infection. **a**, Schematic of the experimental design. **b**, Levels of subgenomic viral transcripts, determined by qRT-PCR, in hPSC-derived AWOs, ALOs and CMs at 48 h.p.i. with SARS-CoV-2 at different m.o.i. (m.o.i. = 0.01, 0.10 and 1.00). The dashed red line indicates the detection limit. **c**, Three-dimensional analysis of transcriptional changes in hPSC-derived AWOs, ALOs and CMs infected at 48 h.p.i. (m.o.i. = 0.01, 0.10 and 1.00). The genes that were significantly changed (log_2_(fold change) > 0.75, base mean > 10 and adjusted *P* < 0.05) in each condition are highlighted in purple. **d**, Heatmap of the protein-coding genes that were increased for at least seven of nine conditions in hPSC-derived AWOs, ALOs and CMs at 48 h.p.i. (m.o.i. = 0.01, 0.10 and 1.00). **c**,**d**, Data are presented as an integration of all biological replicates. **e**,**f**, Representative confocal images (**e**) and the calculated percentage of SARS-N^+^ cells in the cTnT^+^ subpopulation (**f**) of hPSC-CMs infected with lentivirus carrying Cas9 and sgRNAs targeting hit genes. *P* values were calculated using an unpaired two-tailed Student’s *t*-test. The red text and red bar highlight that knockout of CIART showed the greatest resistance to SARS-CoV-2 infection. **b**,**e**,**f**, Data are the mean ± s.d. **b**,**c**–**f**, *n* = 3 independent biological replicates. Source data

**Extended Data Fig. 1:**
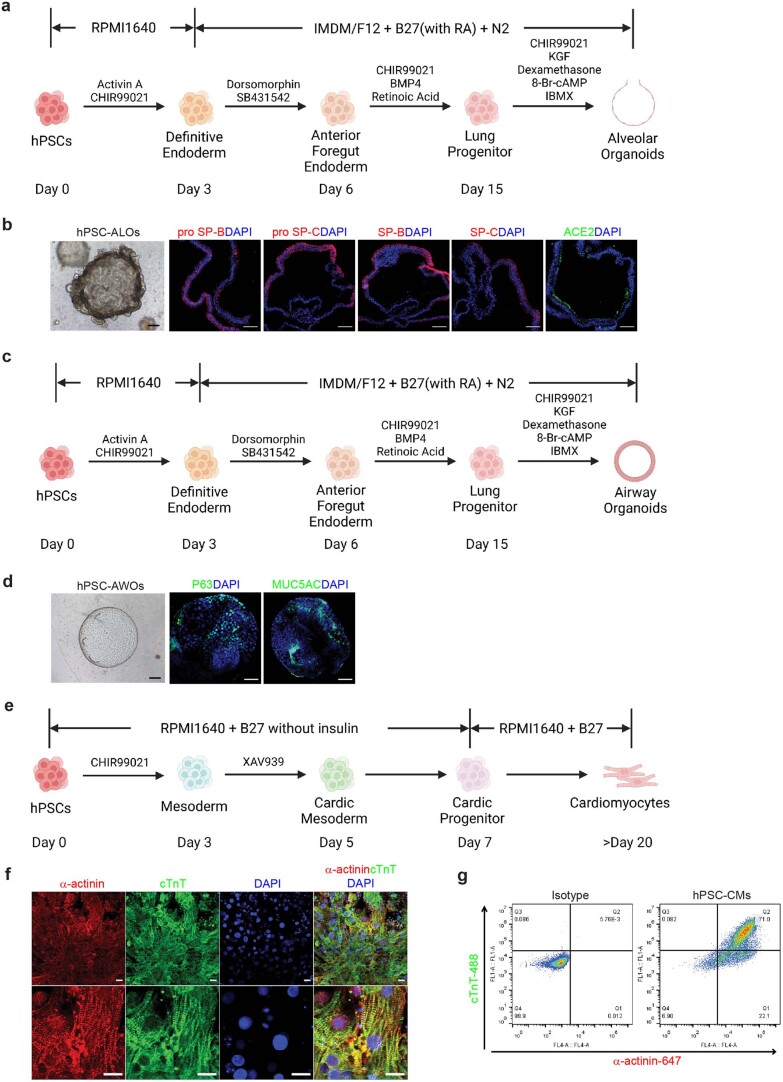
Characterization of hPSC-ALOs, hPSC-AWOs and hPSC-CMs. **a**, Scheme of hPSC-ALO differentiation. **b**, Representative confocal images of day 45 hPSC-ALOs. Scale bar, 100 μm. **c**, Scheme of hPSC-AWO differentiation. **d**, Representative confocal images of day 40 hPSC-AWOs. Scale bar, 100 μm. **e**, Scheme of hPSC-CMs differentiation. **f**, Representative confocal images of day 30 hPSC-CMs. Scale bar, 100 μm. **g**, Flow cytometry analysis of α-actinin and cTnT expression in day 30 hPSC-CMs. Source data

**Extended Data Fig. 2:**
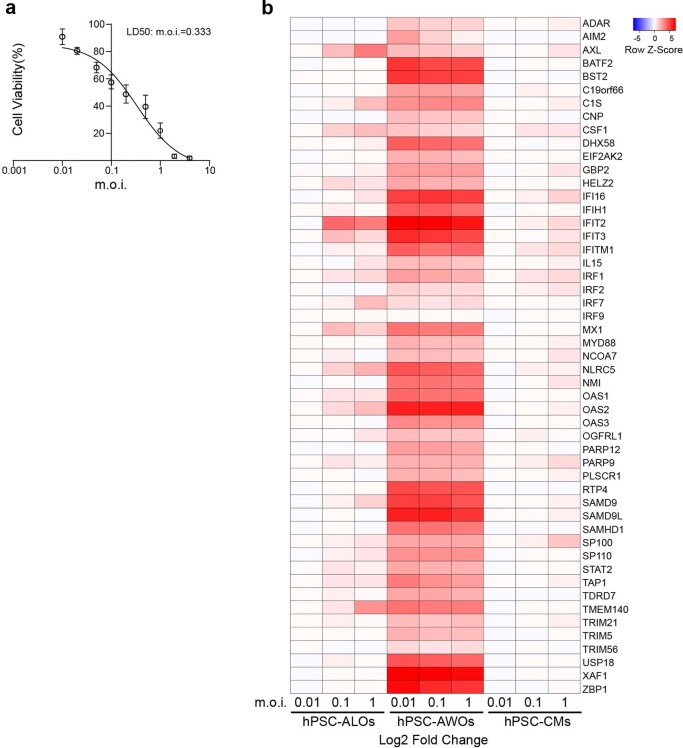
LD_50_ of hPSC-CMs and heatmap of *IFN-1* genes. **a**, Cell viability of hPSC-CMs at 48 h.p.i. with SARS-CoV-2 at different m.o.i. LD_50_, m.o.i. = 0.333. *n* = 3 independent biological replicates. Data are presented as the mean ± s.d. **b**, Heatmap showing induction of genes involved in the type 1 IFN signalling pathway in SARS-CoV-2-infected hPSC-ALOs, -AWOs and -CMs (m.o.i. = 0.01, 0.1 and 1). *n* = 3 independent biological replicates. Data are presented as an integration of all biological replicates. Source data

To profile the relative changes in transcript patterns across cell types, hPSC-derived lung alveolar organoids (ALOs; Extended Data Fig. 1a,b), lung airway organoids (AWOs; Extended Data Fig. 1c,d) and cardiomyocytes (CMs; Extended Data Fig. 1e–g) were each exposed to SARS-CoV-2 at different multiplicities of infection (m.o.i. = 0.01, 0.10 and 1.00; Fig. 1a). To validate this range, we monitored the survival of SARS-CoV-2-infected hPSC-CMs (m.o.i. ranging from 0.01 to 4) at 48 h post infection (h.p.i.) and determined that the median lethal dose (LD_50_) is m.o.i. = 0.333 (Extended Data Fig. 2a). At 48 h.p.i., quantitative real-time PCR (qRT-PCR) determination of SARS-CoV-2 viral subgenomic RNA confirmed robust SARS-CoV-2 infection in hPSC-ALOs (m.o.i. = 0.1 and 1), hPSC-AWOs (all m.o.i.) and hPSC-CMs (all m.o.i.), with relatively higher expression levels in hPSC-AWOs and hPSC-CMs compared with hPSC-ALOs (Fig. 1b). RNA sequencing (RNA-seq) was used to systematically profile transcriptional changes of these hPSC-derived cells/organoids caused by SARS-CoV-2 infection. Three criteria were applied to choose the most highly expressed genes that were significantly changed in each condition: log_2_(fold change) > 0.75, base mean > 10 and adjusted *P* value < 0.05 (Fig. 1c). We first monitored the induction of genes involved in interferon I (IFN-I) pathways following SARS-CoV-2 infection. IFN-I-associated genes were mainly upregulated in hPSC-AWOs but not hPSC-ALOs or hPSC-CMs (Extended Data Fig. 2b). This is consistent with previous findings that the expression pattern of IFN-associated genes differs at distinct respiratory tract sites following SARS-CoV-2 infection^30^. SARS-CoV-2 infection can upregulate host genes, including *ACE2*, to facilitate its infection^31^. Given that we aimed to identify genes that are consistently upregulated following SARS-CoV-2 infection among different organoids/cell types, the IFN-I-associated genes do not meet this criterion. Eighteen genes were identified as significantly changed in seven of nine conditions, excepting the lowest m.o.i. for hPSC-ALOs and in some cases for the lowest m.o.i. for hPSC-CMs (Fig. 1d). These genes (*ARRDC3*, *CIART*, *CSRNP1*, *EGR1*, *FAP*, *FOS*, *H2AC6*, *H2BC5*, *HIVEP2*, *JUN*, *MXD1*, *NR1D1*, *NR4A1*, *NR4A3*, *PPP1R15A*, *SOCS3*, *TIPARP* and *ZNF844*) were individually tested to determine their relevance to infection efficiency. For this purpose, clustered regularly interspaced short palindromic repeats (CRISPR)-based knockout of each gene was performed in hPSC-CMs, which are relatively homogeneous. Briefly, hPSC-CMs were infected with lentivirus expressing Cas9 and two single-guide RNAs (sgRNAs) targeting each gene (Supplementary Table 1). After 3 d of puromycin selection, the knockout hPSC-CMs were subjected to infection with SARS-CoV-2. Knockout of *CIART* showed the greatest resistance to infection compared with the control hPSC-CMs (no sgRNA or scrambled sgRNA), although significantly reduced levels of SARS-CoV-2 infection were also observed by individually targeting *EGR1*, *FOS*, *H2AC6*, *H2BC5*, *JUN*, *MXD1*, *NR1D1*, *NR4A1*, *NR4A3*, *PPP1R15A*, *SOCS3* and *TIPARP* (Fig. 1e,f).

**Extended Data Fig. 3:**
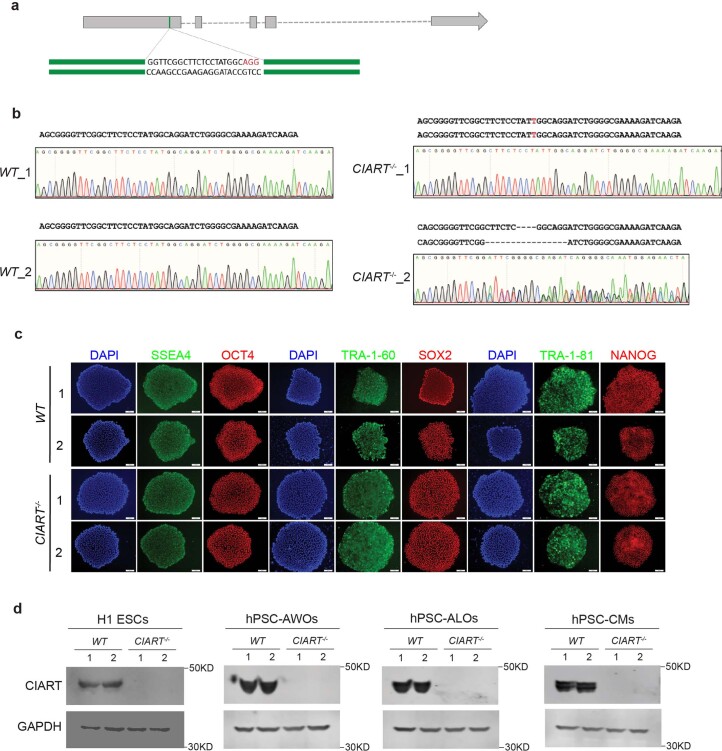
Characterization of WT and *CIART*^−/−^ hPSCs. **a**, Scheme of gene targeting. **b**, DNA sequencing of WT and *CIART*^−/−^ hPSCs. **c**, Immunostaining of pluripotency markers of WT and *CIART*^−/−^ hPSCs. Scale bar, 100 μm. **d**, Western blotting for CIART in lysates from WT and *CIART*^−/−^ H1 ESCs, hPSC-AWOs, hPSC-ALOs and hPSC-CMs. Source data

To validate the requirement for *CIART* in SARS-CoV-2 infections, we used CRISPR–Cas9-based gene targeting to create isogenic *CIART*^−/−^ hPSCs. H1 human embryonic stem cells (H1-hESCs) were electroporated with a vector expressing Cas9 and a specific sgRNA targeting the first exon of *CIART* (Extended Data Fig. 3a). After subcloning, multiple independent clones with biallelic frameshift mutations were expanded. To account for possible variation between different clones, two wild-type (WT) clones (derived from the targeting process but without mutations in *CIART*) and two *CIART*^−/−^ clones (no. 1 and no. 2) were chosen for further analysis. Biallelic indel mutations in each isogenic *CIART*^−/−^ H1-hESC line were verified by DNA sequencing (Extended Data Fig. 3b). Mutant clone no. 1 has a single T insertion at both alleles, whereas mutant clone no. 2 has two distinct deletions. Both indel mutations create early frameshifts that are predicted to generate null alleles. All established clones displayed typical hPSC colony morphology and expressed pluripotency markers, including OCT4, NANOG, SOX2, SSEA4, TRA-1-60 and TRA-1-81 (Extended Data Fig. 3c). Western blotting further validated the knockout of *CIART* in the mutant H1-hESC clones as well as in hPSC-AWOs, hPSC-ALOs and hPSC-CMs (Extended Data Fig. 3d).

**Fig. 2:**
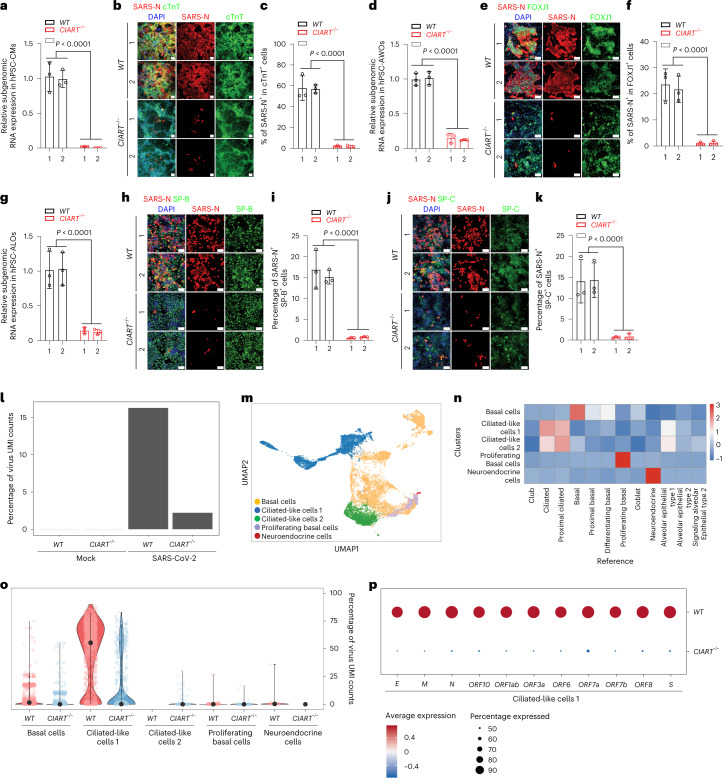
Loss of CIART decreases SARS-CoV-2 infection. **a**, Relative expression levels of viral RNA in WT and *CIART*^−/−^ hPSC-CMs at 24 h.p.i. with SARS-CoV-2 (m.o.i. = 0.3). **b**,**c**, Representative confocal images (**b**) and calculated percentages (**c**) of SARS-N^+^ cells within the cTnT^+^ cell populations of WT and *CIART*^−/−^ hPSC-CMs at 24 h.p.i. with SARS-CoV-2 (m.o.i. = 0.3). **d**, Relative expression levels of viral RNA in WT and *CIART*^−/−^ hPSC-AWOs at 24 h.p.i. with SARS-CoV-2 (m.o.i. = 0.3). **e**,**f**, Representative confocal images (**e**) and calculated percentages (**f**) of SARS-N^+^ cells within the FOXJ1^+^ cell populations of WT and *CIART*^−/−^ hPSC-AWOs at 24 h.p.i. with SARS-CoV-2 (m.o.i. = 0.3). **g**, Relative expression levels of viral RNA in WT and *CIART*^−/−^ hPSC-ALOs at 24 h.p.i. with SARS-CoV-2 (m.o.i. = 0.3). **h**,**i**, Representative confocal images (**h**) and calculated percentages (**i**) of SARS-N^+^ cells in the mature SP-B^+^ cell populations of WT and *CIART*^−/−^ hPSC-ALOs at 24 h.p.i. with SARS-CoV-2 (m.o.i. = 0.3). **j**,**k**, Representative confocal images (**j**) and calculated percentages (**k**) of SARS-N^+^ cells in the mature SP-C^+^ cell populations of WT and *CIART*^−/−^ hPSC-ALOs at 24 h.p.i. with SARS-CoV-2 (m.o.i. = 0.3). **a**–**k**, *n* = 3 independent biological replicates. **l**, Percentage of viral UMI counts in mock- and SARS-CoV-2-infected WT and *CIART*^−/−^ hPSC-AWOs (m.o.i. = 0.1; 24 h.p.i.). **m**, Uniform manifold approximation and projection (UMAP) plot illustrating five cell clusters in the hPSC-AWOs. **n**, Correlation analysis of cell clusters in hPSC-AWOs and adult human lung. **o**, Percentage of viral UMI counts in each cluster of WT and *CIART*^−/−^ hPSC-AWOs following SARS-CoV-2 infection (m.o.i. = 0.1; 24 h.p.i.). **p**, Levels of SARS-CoV-2 viral transcripts in ciliated-like cells 1 of WT and *CIART*^−/−^ hPSC-AWOs following SARS-CoV-2 infection (m.o.i. = 0.1; 24 h.p.i.). Data are the mean ± s.d. *P* values were calculated using an unpaired two-tailed Student’s *t*-test. Scale bars, 100 μm. Source data

**Extended Data Fig. 4:**
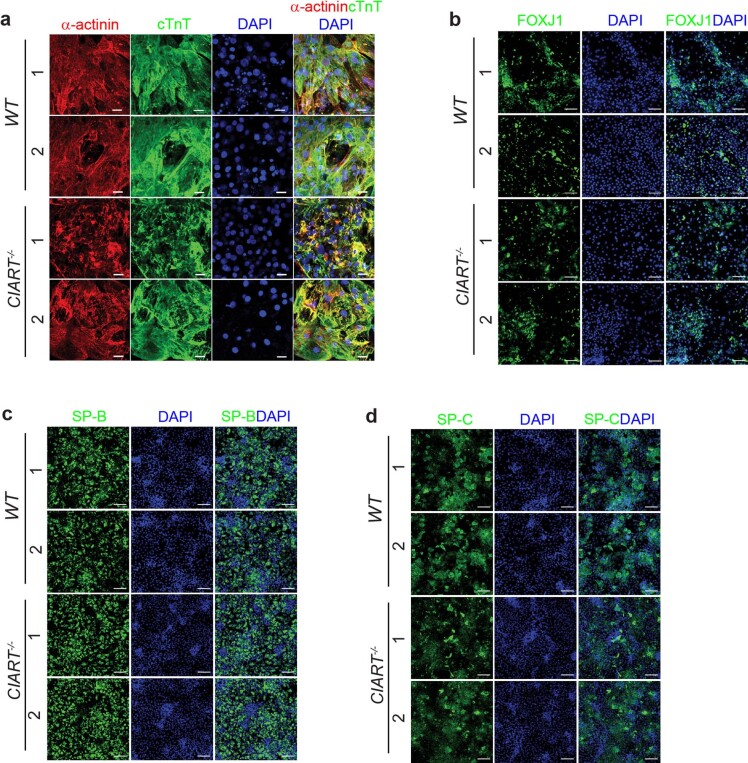
Differentiation of WT and *CIART*^−/−^ hPSCs. **a**, Representative confocal images of α-actinin^+^cTnT^+^ cells of WT and *CIART*^−/−^ hPSC-CMs. Scale bar, 100 μm. **b**, Representative confocal images of FOXJ1^+^ cells of WT and *CIART*^−/−^ hPSC-AWOs. Scale bar, 100 μm. **c**, Representative confocal images of mature SP-B^+^ cells of WT and *CIART*^−/−^ hPSC-ALOs. Scale bar, 100 μm. **d**, Representative confocal images of mature SP-C^+^ cells of WT and *CIART*^−/−^ hPSC-ALOs. Scale bar, 100 μm.

**Extended Data Fig. 5:**
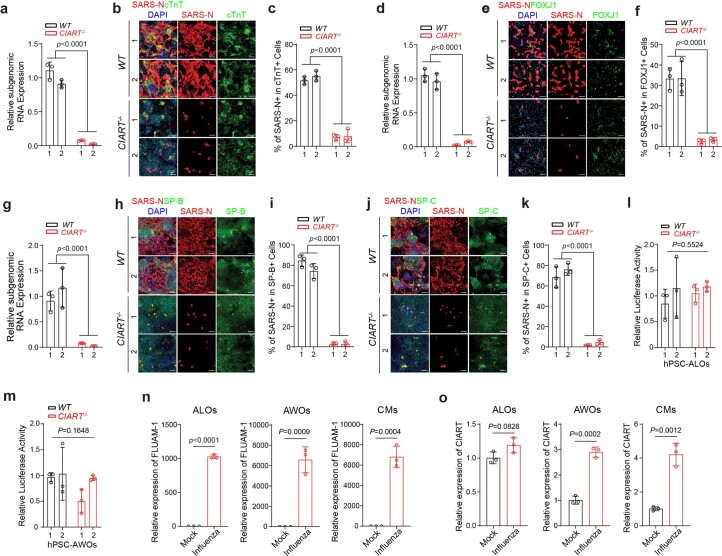
Loss of CIART decreases SARS-CoV-2 infection at 48 h.p.i. **a**, Relative SARS-CoV-2 viral RNA expression levels (at 48 h.p.i.) of WT and *CIART*^−/−^ hPSC-CMs infected with SARS-CoV-2 (m.o.i. = 0.3). *n* = 3 independent biological replicates. **b**,**c**, Representative confocal images (**b**) and quantification (**c**) of SARS-N^+^ cells in cTnT^+^ cells (at 48 h.p.i.) of WT and *CIART*^−/−^ hPSC-CMs infected with SARS-CoV-2 virus (m.o.i. = 0.3). Scale bar, 100 μm. *n* = 3 independent biological replicates. **d**, Relative SARS-CoV-2 viral RNA expression levels at 48 h.p.i. in WT and *CIART*^−/−^ hPSC-AWOs infected with SARS-CoV-2 (m.o.i. = 0.3). *n* = 3 independent biological replicates. **e**,**f**, Representative confocal images (**e**) and quantification (**f**) of SARS-N^+^ cells in FOXJ1^+^ cells (at 48 h.p.i.) of WT and *CIART*^−/−^ hPSC-AWOs infected with SARS-CoV-2 (m.o.i. = 0.3). Scale bar, 100 μm. *n* = 3 independent biological replicates. **g**, Relative SARS-CoV-2 viral RNA expression levels (at 48 h.p.i.) of WT and *CIART*^−/−^ hPSC-ALOs infected with SARS-CoV-2 (m.o.i. = 0.3). *n* = 3 independent biological replicates. **h**,**i**, Representative confocal images (**h**) and quantification (**i**) of SARS-N^+^ cells in mature SP-B^+^ cells (at 48 h.p.i.) of WT and *CIART*^−/−^ hPSC-ALOs infected with SARS-CoV-2 (m.o.i. = 0.3). Scale bar, 100 μm. *n* = 3 independent biological replicates. **j**,**k**, Representative confocal images (**j**) and quantification (**k**) of SARS-N^+^ cells in mature SP-C^+^ cells (at 48 h.p.i.) of WT and *CIART*^−/−^ hPSC-ALOs infected with SARS-CoV-2 (m.o.i. = 0.3). Scale bar, 100 μm. *n* = 3 independent biological replicates. **l**, Relative luciferase activity (at 24 h.p.i.) for hPSC-ALOs infected with SARS-CoV-2 pseudo-typed entry reporter virus (m.o.i. = 0.01). *n* = 3 independent biological replicates. **m**, Relative luciferase activity (at 24 h.p.i.) for hPSC-AWOs infected with SARS-CoV-2 pseudo-typed entry reporter virus (m.o.i. = 0.01). *n* = 3 independent biological replicates. **n**, Relative influenza viral RNA expression level of mock- and influenza-infected hPSC-ALOs, -AWOs and -CMs at 48 h.p.i. (m.o.i. = 1). *n* = 3 independent biological replicates. **o**, Relative *CIART* expression level of mock- and influenza-infected hPSC-ALOs, -AWOs and -CMs at 48 h.p.i. (m.o.i. = 1). *n* = 3 independent biological replicates. Data are presented as the mean ± s.d. *P* values were calculated using an unpaired two-tailed Student’s *t*-test. Source data

The isogenic WT and mutant *CIART*^−/−^ hESC lines were differentiated to CMs, AWOs and ALOs to evaluate the impact of *CIART* in SARS-CoV-2 infection. Both WT and *CIART*^−/−^ H1-hESCs showed comparable capacities to differentiate into CMs, identified by signature sarcomere structures and expression of the cardiac marker α-actinin and cardiac Troponin-T (cTnT; Extended Data Fig. 4a). At 24 h.p.i., the *CIART*^−/−^ hPSC-CMs were highly resistant to viral infection, as determined by qRT-PCR (Fig. 2a) and immunofluorescence staining analyses (Fig. 2b,c). The *CIART*^−/−^ and WT hPSCs were also equally capable of differentiating to hPSC-AWOs, based on the generation of FOXJ1^+^ ciliated cells (Extended Data Fig. 4b). Similar to mutant CMs, significantly decreased levels of SARS-CoV-2 infection were detected in *CIART*^−/−^ hPSC-AWOs, as determined by qRT-PCR (Fig. 2d), whereas the percentages of SARS-N^+^ cells were significantly lower in FOXJ1^+^ ciliated cells of *CIART*^−/−^ hPSC-AWOs compared with the WT hPSC-AWOs (Fig. 2e,f). Finally, *CIART*^−/−^ hPSCs were differentiated to hPSC-ALOs. Both WT and *CIART*^−/−^ cells generated hPSC-ALOs with abundant mature SP-B and mature SP-C double-positive (SP-B^+^SP-C^+^) alveolar type 2 cells (Extended Data Fig. 4c,d). As seen in hPSC-CMs and hPSC-AWOs, *CIART*^−/−^ hPSC-ALOs were highly resistant to infection, as determined by qRT-PCR (Fig. 2g) and immunofluorescence staining in SP-B^+^SP-C^+^ alveolar type 2 cells (Fig. 2h–k). Consistent with the results at 24 h.p.i., *CIART*^−/−^ hPSC-CMs (Extended Data Fig. 5a–c), hPSC-AWOs (Extended Data Fig. 5d–f) and hPSC-ALOs (Extended Data Fig. 5g–k) also showed significantly reduced SARS-CoV-2 infection compared with their WT counterparts at 48 h.p.i. To determine whether loss of *CIART* affects viral entry, *CIART*^−/−^ hPSC-AWOs and hPSC-ALOs were infected with a SARS-CoV-2 Spike protein pseudo-typed entry virus. No significant difference in viral entry was detected (Extended Data Fig. 5l,m). However, it is worth noting that the overall infection rate of pseudo-typed entry viruses in primary cells/organoids can be relatively low. Together, the data suggest that loss of *CIART* impairs SARS-CoV-2 infection through an entry-independent mechanism. We infected hPSC-ALOs, -AWOs and -CMs with influenza to test whether *CIART* upregulation is specific to SARS-CoV-2 infection. Robust influenza infection was determined by qRT-PCR (Extended Data Fig. 5n). *CIART* expression was also upregulated following influenza infection of ALOs, AWOs and CMs (Extended Data Fig. 5o), indicating that the induction of CIART is not specific to SARS-CoV-2 infection.

**Extended Data Fig. 6:**
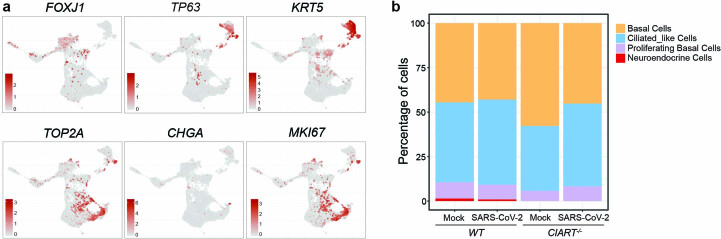
scRNA-seq analysis of mock- and SARS-CoV-2-infected WT and *CIART*^−/−^ hPSC-AWOs. **a**, UMAP of marker genes in hPSC-AWOs. **b**, Percentage of each cell type in mock- and SARS-CoV-2-infected WT and *CIART*^−/−^ hPSC-AWOs.

Lung organoids contain multiple cell types, so single-cell RNA-seq (scRNA-seq) was applied to analyse SARS-CoV-2 infection in defined lineages of hPSC-AWOs. Both WT and *CIART*^−/−^ AWOs were infected with SARS-CoV-2 (m.o.i. = 0.1) and dissociated into single cells for scRNA-seq at 24 h.p.i. Consistent with previous observations (Fig. 2d), the percentage of viral reads in infected *CIART*^−/−^ AWOs was significantly lower compared with WT AWOs (Fig. 2l). Five clusters were identified, including basal cells (cluster 0), ciliated-like cells (clusters 1 and 2), proliferating basal cells (cluster 3) and neuroendocrine cells (cluster 4; Fig. 2m and Extended Data Fig. 6a,b). Correlation analysis of signature genes further validated the identity of the hPSC-derived ciliated-like cell population, showing high similarity to adult human ciliated cells^32^ (Fig. 2n). We examined the percentage of viral unique molecular identifier (UMI) counts in each cluster of WT and *CIART*^−/−^ AWOs following SARS-CoV-2 infection. Viral UMIs were mainly detected in ciliated-like cell cluster 1 (Fig. 2o). The percentage of viral UMIs was significantly lower in each cluster of *CIART*^−/−^ AWOs compared with their WT AWO counterparts (Fig. 2o). We further examined individual viral transcripts in ciliated-like cells and found that all viral transcripts were significantly decreased in *CIART*^−/−^ ciliated-like cells (Fig. 2p). Together, these data further confirm that loss of *CIART* significantly impairs SARS-CoV-2 infection.

**Fig. 3:**
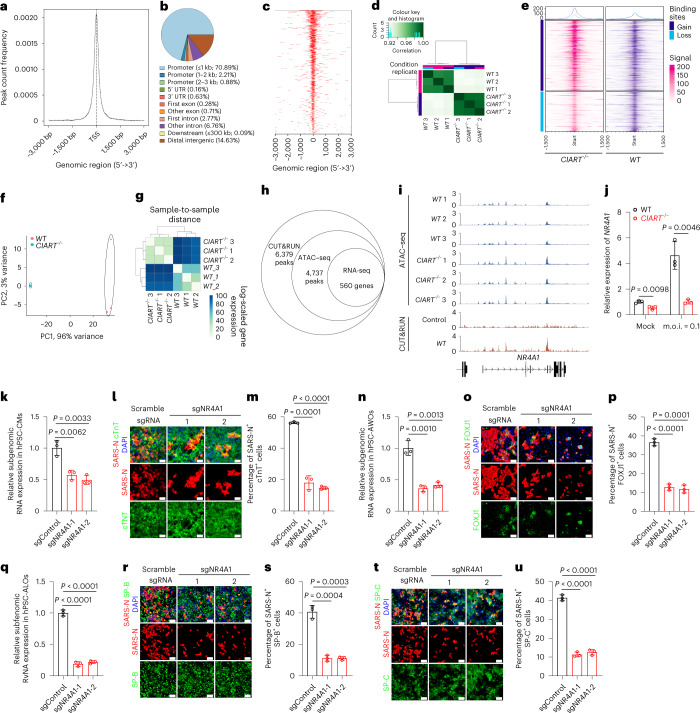
CIART promotes SARS-CoV-2 infection through NR4A1 regulation. **a**, Average profile of *CIART* CUT&RUN peaks in WT hPSC-CMs around the TSS. **b**, Distribution of the genomic locations of CUT&RUN peaks in WT hPSC-CMs. **c**, Profile heatmap showing the distribution of CUT&RUN peaks in WT hPSC-CMs around the TSS. **a**–**c**, Data are presented as an integration of all biological replicates; *n* = 2 independent biological replicates. **d**, Clustering analysis of ATAC-seq data of WT and *CIART*^−/−^ hPSC-CMs. **e**, Profile ATAC-seq heatmap showing the enrichment of gain and loss sites in WT and *CIART*^−/−^ hPSC-CMs. **f**,**g**, RNA-sequencing PCA (**f**) and sample clustering (**g**) analysis of WT and *CIART*^−/−^ hPSC-AWOs under mock infection conditions. **d**–**g**, Data are presented as the individual biological replicates (**d**,**f**,**g**) or an integration of all biological replicates (**e**); *n* = 3 independent biological replicates. **h**, Summary of peaks in the CUT&RUN, ATAC-seq and RNA-seq assays. **i**, Peaks associated with the *NR4A1* gene in WT and *CIART*^−/−^ hPSC-CMs in the ATAC-seq and CUT&RUN assays. Data are presented as individual biological replicates (*n* = 3 for ATAC-seq) and 2 for CUT&RUN). The schematic below the plots illustrates the exon and intron regions of gene NR4A1 in the genome. **j**, Expression levels, measured by qRT-PCR, of *NR4A1* in WT and *CIART*^−/−^ hPSC-CMs following mock or SARS-CoV-2 infection (m.o.i. = 0.1). **k**–**m**, Relative expression levels of SARS-CoV-2 RNA in hPSC-CMs (**k**) as well as representative confocal images (**l**) and calculated percentages (**m**) of SARS-N^+^ cells in the cTnT^+^ subsets of hPSC-CMs expressing scramble sgRNA or sgNR4A1 at 24 h.p.i. (m.o.i. = 0.1). **n**–**p**, Relative expression levels of SARS-CoV-2 viral RNA in hPSC-AWOs (**n**) as well as representative confocal images (**o**) and calculated percentages (**p**) of SARS-N^+^ cells within the FOXJ1^+^ cell populations of hPSC-AWOs expressing scramble sgRNA or sgNR4A1 at 24 h.p.i. (m.o.i. = 0.1). **q**–**s**. Relative expression levels of SARS-CoV-2 RNA in hPSC-ALOs (**q**) as well as representative confocal images (**r**) and calculated percentages (**s**) of SARS-N^+^ cells within the mature SP-B^+^ cell populations of hPSC-ALOs expressing scramble sgRNA or sgNR4A1 at 24 h.p.i. (m.o.i. = 0.1). **t**,**u**, Representative confocal images (**t**) and calculated percentages (**u**) of SARS-N^+^ cells within mature SP-C^+^ cell populations of hPSC-ALOs expressing scramble sgRNA or sgNR4A1 at 24 h.p.i. (m.o.i. = 0.1). **j**–**u**, Data are presented as the mean ± s.d. (**j**,**k**,**m**,**n**,**p**,**q**,**s**,**u**); *n* = 3 independent biological replicates. *P* values were calculated by unpaired two-tailed Student’s *t*-test. Scale bars, 100 μm. Source data

*CIART* was identified as a key transcription factor involved in the regulation of the mammalian circadian clock^26–28^. We performed cleavage under targets and release using nuclease (CUT&RUN) chromatin profiling, assay for transposase-accessible chromatin using sequencing (ATAC-seq) and RNA-seq to identify downstream targets and signalling pathways regulated by *CIART* in hPSC-CMs. We identified 6,379 peaks using CUT&RUN, most of which were close to the transcription start site (TSS; Fig. 3a,b). More than 50% of the identified peaks were located in proximal promoter regions (Fig. 3c). The ATAC-seq peaks derived from WT and *CIART*^−/−^ hPSC-CMs clustered separately (Fig. 3d) with 12,529 sites gained and 564 sites lost in *CIART*^−/−^ hPSC-CMs compared with WT hPSC-CMs (Fig. 3e). From the RNA-seq data, clustering and principal component analyses (PCA), we confirmed that WT and *CIART*^−/−^ hPSC-CMs are distinguishable at the transcript level (Fig. 3f,g). When we combined the CUT&RUN, ATAC-seq and RNA-seq assays, 671 peaks (associated with 560 genes) that are bound by CIART in WT hPSC-CMs and are significantly changed between WT and *CIART*^−/−^ hPSC-CMs at both transcriptional and chromatin accessibility levels were identified (Fig. 3h). Interestingly, one of these genes, *NR4A1*, was also identified along with *CIART* as an upregulated hit gene following SARS-CoV-2 infection in the initial hPSC-derived multi-organoid platform (Fig. 1f). The peaks associated with *NR4A1* in the CUT&RUN assay (Fig. 3i) were correlated in the ATAC-seq data and significantly changed in *CIART*^−/−^ hPSC-CMs compared with WT hPSC-CMs. Interestingly, some ATAC-seq peaks were enhanced in the mutant cells, suggesting an altered chromatin structure with loss of CIART binding. Finally, qRT-PCR assays confirmed that the transcript levels of *NR4A1* were significantly decreased in both mock- and SARS-CoV-2-infected *CIART*^−/−^ hPSC-CMs (Fig. 3j), consistent with the RNA-seq data. To determine whether *NR4A1* impacts SARS-CoV-2 infection, hPSC-derived CMs, AWOs and ALOs were infected with lentivirus expressing Cas9 and one of two different sgRNAs targeting *NR4A1* (Supplementary Table 1). CMs, AWOs and ALOs expressing sgRNA targeting *NR4A1* (sgNR4A1) or scrambled sgRNA control were infected with SARS-CoV-2 (m.o.i. = 0.1). At 24 h.p.i., both subgenomic viral RNAs and the percentage of SARS-N^+^ cells in cTnT^+^ CMs (Fig. 3k–m), FOXJ1^+^ cells in AWOs (Fig. 3n–p) and SP-B^+^SP-C^+^ cells in ALOs (Fig. 3q–u) were significantly decreased in cells expressing sgNR4A1 compared with those expressing scramble sgRNA.

**Fig. 4:**
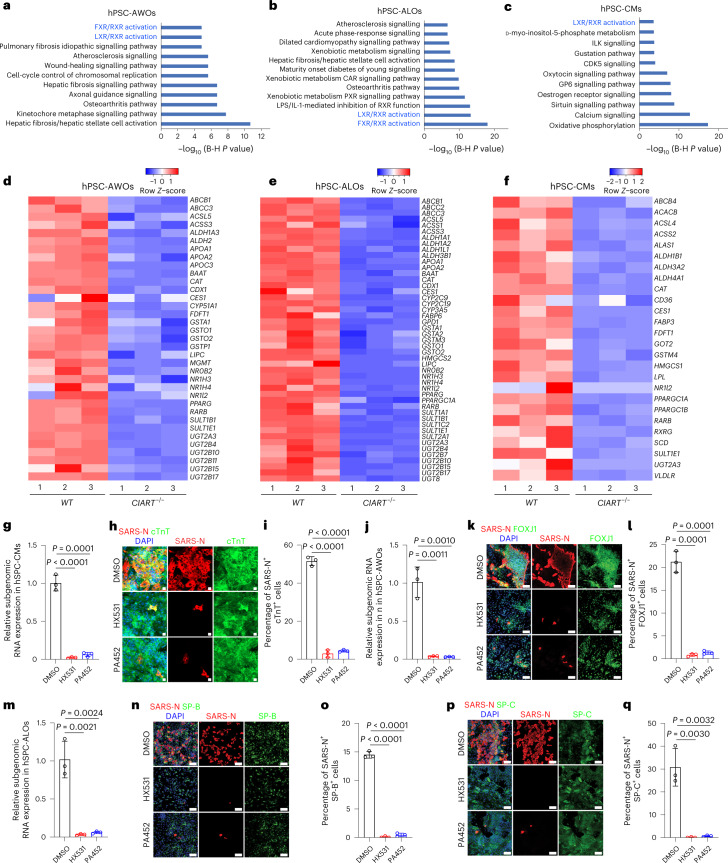
CIART regulates SARS-CoV-2 infection through the RXR pathway. **a**–**c**, Enriched pathways in *CIART*^−/−^ hPSC-AWOs (**a**), hPSC-ALOs (**b**) and hPSC-CMs (**c**) relative to their WT counterparts. The B-H p-value was corrected by the Benjamini-Hochberg method. The text in blue highlights that the RXR signaling pathway was enriched in *CIART*^−/−^ AWOs, ALOs and CMs. **d**–**f**, Heatmap of RXR pathway-associated genes comparing WT and *CIART*^−/−^ hPSC-AWOs (**d**), hPSC-AWOs (**e**) and hPSC-CMs (**f**). **g**, Relative expression levels of SARS-CoV-2 RNA in hPSC-CMs treated with DMSO, HX531 or PA452 (24 h.p.i.; m.o.i. = 0.3). **h**,**i**, Representative confocal images (**h**) and calculated percentages (**i**) of SARS-N^+^ cells in the cTnT^+^ subsets of hPSC-CMs treated with DMSO, HX531 or PA452 (24 h.p.i.; m.o.i. = 0.3). **j**, Relative expression levels of SARS-CoV-2 RNA in hPSC-AWOs treated with DMSO, HX531 or PA452 (24 h.p.i.; m.o.i. = 0.3). **k**,**l**, Representative confocal images (**k**) and calculated percentages (**l**) of SARS-N^+^ cells within the FOXJ1^+^ cell populations of hPSC-AWOs treated with DMSO, HX531 or PA452 (24 h.p.i.; m.o.i. = 0.3). **m**, Relative expression levels of SARS-CoV-2 RNA in hPSC-ALOs treated with DMSO, HX531 or PA452 (24 h.p.i.; m.o.i. = 0.3). **n**,**o**, Representative confocal images (**n**) and calculated percentages (**o**) of SARS-N^+^ cells within the mature SP-B^+^ cell populations of hPSC-ALOs treated with DMSO, HX531 or PA452 (24 h.p.i.; m.o.i. = 0.3). **p**,**q**, Representative confocal images (**p**) and calculated percentages (**q**) of SARS-N^+^ cells within the mature SP-C^+^ cell populations of hPSC-ALOs treated with DMSO, HX531 or PA452 (24 h.p.i.; m.o.i. = 0.3). Data are presented as an integration of all biological replicates (**a**–**c**), individual biological replicates (**d**–**f**) or the mean ± s.d. (**g**,**i**,**j**,**l**,**m**,**o**,**q**); *n* = 3 independent biological replicates. *P* values were calculated using a paired or an unpaired two-tailed Student’s *t*-test. Scale bars, 100 μm. Source data

**Extended Data Fig. 7:**
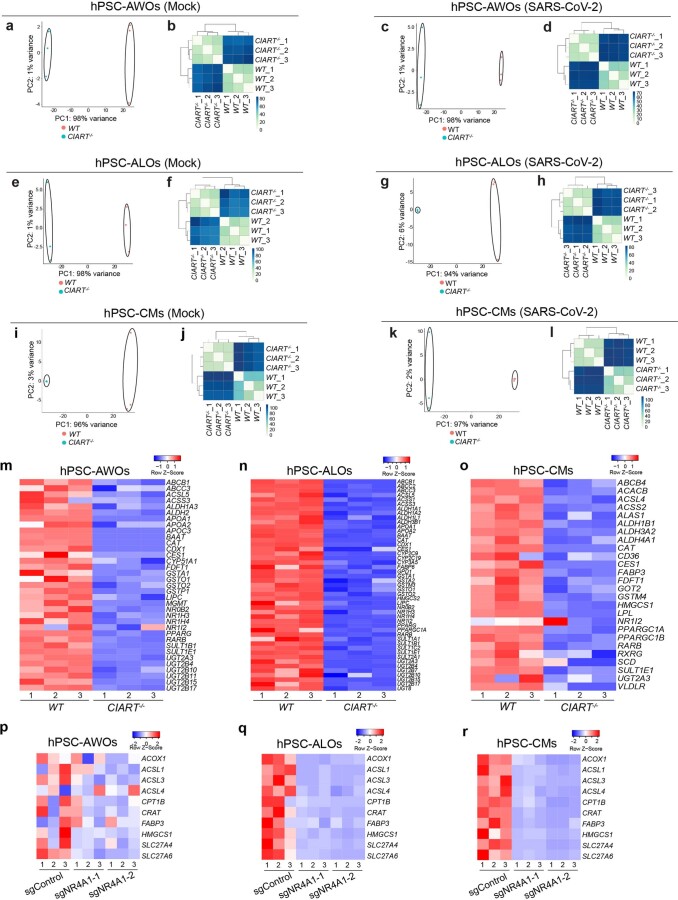
RNA-seq analysis of WT and *CIART*^−/−^ hPSC-derived AWOs, ALOs and CMs. **a**,**b**, PCA (**a**) and sample clustering (**b**) analysis of WT and *CIART*^−/−^ hPSC-AWOs under mock infection conditions. **c,d**, PCA (**c**) and sample clustering (**d**) analysis of WT and *CIART*^−/−^ hPSC-AWOs at 24 h.p.i. (m.o.i. = 0.1). **e**,**f**, PCA (**e**) and sample clustering (**f**) analysis of WT and *CIART*^−/−^ hPSC-ALOs under mock infection conditions. **g**,**h**, PCA (**g**) and sample clustering (**h**) analysis of WT and *CIART*^−/−^ hPSC-ALOs at 24 h.p.i. (m.o.i. = 0.1). **i**,**j**, PCA (**i**) and sample clustering (**j**) analysis of WT and *CIART*^−/−^ hPSC-CMs under mock infection conditions. **k**,**l**, PCA (**k**) and sample clustering (**l**) analysis of WT and *CIART*^−/−^ hPSC-CMs at 24 h.p.i. (m.o.i. = 0.1). **m**–**o**, Heatmaps of RXR pathway-associated genes in WT and *CIART*^−/−^ hPSC-derived AWOs (**m**), ALOs (**n**) and CMs (**o**) at 24 h.p.i. (m.o.i. = 0.1). *n* = 3 independent biological replicates. Data are presented as individual biological replicates. **p**–**r**, Heatmap of RXR pathway-associated genes in control and sgNR4A1-infected hPSC-derived AWOs (**p**), ALOs (**q**) and CMs (**r**). *n* = 3 independent biological replicates. Data are presented as individual biological replicates. Source data

To define the downstream signalling pathways regulated by *CIART*, RNA-seq was applied to analyse WT as well as *CIART*^−/−^ hPSC-AWOs, hPSC-ALOs and hPSC-CMs. Clustering and PCA analyses showed that the transcript profiles of WT and *CIART*^−/−^ hPSC-AWOs clustered separately both in mock conditions (PCA plot, Extended Data Fig. 7a; clustering, Extended Data Fig. 7b) and following SARS-CoV-2 infection (PCA plot, Extended Data Fig. 7c; clustering, Extended Data Fig. 7d). Similarly, the transcript profiles of WT and *CIART*^−/−^ hPSC-ALOs clustered separately both in mock conditions (PCA plot, Extended Data Fig. 7e; clustering, Extended Data Fig. 7f) and 24 h.p.i. with SARS-CoV-2 (m.o.i. = 0.1; PCA plot, Extended Data Fig. 7g; clustering, Extended Data Fig. 7h). The transcript profiles of WT and *CIART*^−/−^ hPSC-CMs clustered separately both in mock conditions (PCA plot, Extended Data Fig. 7i; clustering, Extended Data Fig. 7j) and 24 h.p.i. with SARS-CoV-2 (m.o.i. = 0.1; PCA plot, Extended Data Fig. 7k; clustering, Extended Data Fig. 7l). Ingenuity pathway analysis highlighted Retinoid X receptor (RXR) signalling pathways in *CIART*^−/−^ hPSC-AWOs (Fig. 4a), *CIART*^−/−^ hPSC-ALOs (Fig. 4b) and *CIART*^−/−^ hPSC-CMs (Fig. 4c). Heatmaps showed the downregulation of RXR pathway-associated genes in *CIART*^−/−^ hPSC-AWOs (Fig. 4d), hPSC-ALOs (Fig. 4e) and hPSC-CMs (Fig. 4f). In addition, RXR pathway-associated genes were also downregulated following SARS-CoV-2 infection in *CIART*^−/−^ hPSC-AWOs (Extended Data Fig. 7m), hPSC-ALOs (Extended Data Fig. 7n) and hPSC-CMs (Extended Data Fig. 7o). Previous studies reported that NR4A1 could heterodimerize with RXR and increase the potential of RXR to modulate gene expression^33^. The downregulation of RXR pathway-associated genes in hPSC-AWOs (Extended Data Fig. 7p), hPSC-ALOs (Extended Data Fig. 7q) and hPSC-CMs (Extended Data Fig. 7r) expressing sgNR4A1 was confirmed using qRT-PCR assays. Finally, hPSC-AWOs, hPSC-ALOs and hPSC-CMs were treated with RXR inhibitors, followed by SARS-CoV-2 infection. Treatment with the RXR inhibitors HX531 and PA452 blocked SARS-CoV-2 infection in hPSC-CMs (subgenomic viral RNA, Fig. 4g; viral antigen, Fig. 4h,i), hPSC-AWOs (subgenomic viral RNA, Fig. 4j; viral antigen, Fig. 4k,l) and hPSC-ALOs (subgenomic viral RNA, Fig. 4m; viral antigen, Fig. 4n–q) at 24 h.p.i., as determined using qRT-PCR and immunostaining assays. Together, these data confirm that inhibition of the RXR pathway suppresses SARS-CoV-2 infection.

**Extended Data Fig. 8:**
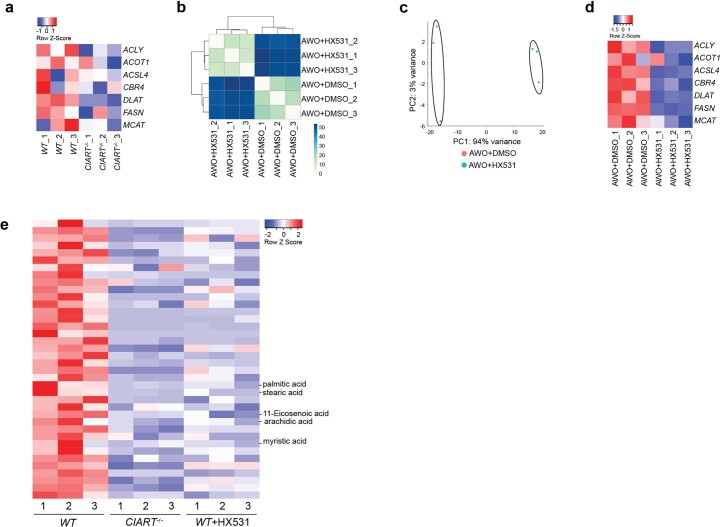
RNA-seq and metabolism profiling of WT and *CIART*^−/−^ hPSC-AWOs. **a**, Heatmap of genes involved in fatty-acid synthesis for WT and *CIART*^−/−^ hPSC-AWOs. *n* = 3 independent biological replicates. **b**, Clustering analysis of DMSO- and HX531-treated hPSC-AWOs. *n* = 3 independent biological replicates. **c**, PCA of DMSO- and HX531-treated hPSC-AWOs. *n* = 3 independent biological replicates. **d**, Heatmap of genes involved in fatty-acid synthesis for DMSO- and HX531-treated WT hPSC-AWOs. *n* = 3 independent biological replicates. **e**, Heatmap of metabolic profiles for WT, *CIART*^−/−^ and HX531-treated hPSC-AWOs. *n* = 3 independent biological replicates. Source data

Previous studies demonstrated that the RXR signalling pathway regulates fatty-acid metabolism^34,35^. We analysed RNA-seq data of WT and *CIART*^−/−^ hPSC-AWOs and found decreased expression levels in *CIART*^−/−^ hPSC-AWOs for multiple genes involved in fatty-acid synthesis including *ACLY*, *ACOT1*, *ACSL4*, *CBR4*, *DLAT*, *FASN* and *MCAT* (Extended Data Fig. 8a). We performed RNA-seq analysis of HX531- or dimethylsulfoxide (DMSO)-treated AWOs. Consistent with the data for *CIART*^−/−^ hPSC-AWOs, the expression of fatty-acid-synthesis-associated genes are downregulated in HX531-treated hPSC-AWOs (clustering, Extended Data Fig. 8b; PCA plot, Extended Data Fig. 8c; heatmap, Extended Data Fig. 8d). To further confirm the change in fatty-acid synthesis caused by *CIART* knockout or the RXR inhibitor HX531, we performed metabolism profiling of WT, *CIART*^−/−^ and WT + HX531-treated hPSC-AWOs. Several fatty acids, including palmitic acid, stearic acid, 11-eicosenoic acid, arachidic acid and myristic acid, were decreased in *CIART*^−/−^ and WT + HX531 hPSC-AWOs compared with the control WT without treatment (Extended Data Fig. 8e). Previous studies reported that inhibition of fatty-acid synthesis could suppress SARS-CoV-2 infection^8,36^. Together, these data suggest that loss of CIART decreases fatty-acid synthesis, which results in diminished SARS-CoV-2 infection.

Several host factors regulating SARS-CoV-2 infection have been identified by CRISPR-based screens using Vero E6 cells, cancer cell lines and patient-derived organoids^37,38^. Here we used the hPSC-derived multi-organoid system (including AWOs, ALOs and CMs). It is technically very challenging to isolate primary adult ALOs, AWOs and CMs from the same donor, and thus hPSCs were an optimal system for our purpose as they can be differentiated into multiple types of isogenic cells and organoids. However, a limitation of hPSC-derived organoids is that they are not as mature as adult tissue. Using the hPSC-derived multi-organoid system, we identified *CIART*, a nuclear transcription factor, as a key regulator of SARS-CoV-2 infection. Compared with control WT cells, isogenic derivatives from *CIART*^−/−^ hPSCs poorly support SARS-CoV-2 infection across multiple lineages including in AWOs, ALOs and CMs. We found that CIART regulates SARS-CoV-2 infection through an entry-independent mechanism. Loss of CIART blocks SARS-CoV-2 infection by downregulating the RXR pathway, at least in part through NR4A1, leading to decreased fatty-acid synthesis, thereby impairing viral infection. Using the multi-organoid platform, we identified a previously unknown role for the CIART–RXR axis in supporting SARS-CoV-2 infection of human primary cells/organoids. CIART was originally identified as a transcriptional repressor that forms a negative regulatory component of the circadian clock^26–28^. Circadian rhythm has been shown to be involved in many biological processes. This study highlights the potential role of the host-cell circadian rhythm on viral infection. Interestingly, studies have been performed to develop RXR modulators as drug candidates^39^, and in that sense, this study could aid in the advancement of strategies for the development of antiviral drugs.

## Methods

All embryonic stem cell studies were approved by the Tri-Institutional ESCRO Committee (Weill Cornell Medicine, Memorial Sloan Kettering Cancer Center and Rockefeller University).

### Cell lines and culture conditions

H1-hESCs (WiCell, WA01) were cultured and maintained in StemFlex medium (Gibco) on 1% Matrigel-coated six-well plates. The medium was changed daily. When the H1-hESCs reached approximately 90% confluency, the cells were passaged at 1:6–1:10 with ReLeSR (Stem Cell Technology). Vero E6 cells (provided by the ATCC, CRL-1586) were cultured in DMEM medium supplemented with 2% fetal bovine serum (FBS), 4.5 g l^−1^ d-glucose, 4 mM l-glutamine, 10 mM non-essential amino acids, 1 mM sodium pyruvate and 10 mM HEPES. HEK 293T cells (provided by the ATCC, CRL-3216) were cultured in DMEM supplemented with 10% FBS. All cell lines were cultured at 37 °C with 5% CO_2_ and were tested for mycoplasma contamination every six months.

### Creation of isogenic H1-hESC lines

CRISPR sgRNA sequences were designed using the web resources available at http://chopchop.cbu.uib.no/. The target sequences are listed in Supplementary Table 1. Each target sequence was cloned into the pSpCas9(BB)-2A-Puro (PX459) V2.0 vector (Addgene, cat. no. 62988) to make the gene-targeting constructs. *CIART*-knockout lines were created based on H1-hESCs. Briefly, H1 cells were dissociated using Accutase (Innovative Cell Technologies) and electroporated (5 × 10^5^ cells per sample) with 4 μg sgRNA-construct plasmids using Human Stem Cell Nucleofector solution (Lonza) following the manufacturer’s instructions. The cells were then seeded into two wells of 24-well plates and cultured in StemFlex medium with 10 μM Y-27632. They were switched to StemFlex medium with 0.5 mg ml^−1^ puromycin the following day and maintained for 2 d. After puromycin selection, hPSCs were dissociated into single cells using Accutase and re-plated at a density of 3 cells per well in 96-well plates. Y-27632 (10 μM) was added to the cells for the first 2 d. After 10 d, individual colonies were picked and re-plated into two wells of 96-well plates. One well of the cells from an individual clone was analysed by DNA sequencing. For biallelic frameshift mutants, we chose homozygous or compound heterozygous mutants. Wild-type lines from the same gene-editing experiment were included as controls to account for potential non-specific effects associated with the gene-targeting process.

### CM differentiation

To derive hPSC-CMs, we used a protocol from a previous study^40^, with slight modifications. The hPSCs were passaged at a density of 3 × 10^5^ cells per well in six-well plates and cultured in a humidified incubator with 5% CO_2_ at 37 °C for 2 d. On day 0 of differentiation, hPSCs with around 90% confluency were treated with RPMI 1640 supplemented with B27 minus insulin (Thermo Fisher) and 6 μM CHIR99021 (Sigma) for 48 h. The medium was replaced with RPMI 1640 supplemented with B27 minus insulin on day 2. On day 3, the medium was changed to RPMI 1640 supplemented with B27 minus insulin and 5 μM XAV939 (Cayman Chemical). On day 5, the medium was switched back to RPMI 1640 supplemented with B27 minus insulin. From day 7, the medium was changed to RPMI 1640 plus normal B27 (Life Technologies); this medium was replaced every 2 d.

### ALO differentiation

The protocol used for the generation of hPSC-ALOs was slightly modified from a previous study^2^. Briefly, hPSCs were differentiated to definitive endoderm with RPMI 1640 supplemented with 100 ng ml^−1^ Activin A and 3 μM CHIR99021 for 1 d, and then RPMI 1640 supplemented with 100 ng ml^−1^ Activin A and 0.2% FBS for another 2 d. Definitive endoderm cells were further cultured in a complete serum-free differentiation medium (cSFDM) consisting of a base medium of 75% IMDM (Thermo Fisher) and 25% Ham’s F12 (Thermo Fisher) with B27 supplement plus retinoic acid (Invitrogen), N2 supplement (Invitrogen), 0.1% bovine serum albumin fraction V (Invitrogen), monothioglycerol (Sigma), GlutaMAX (Thermo Fisher) and ascorbic acid (Sigma). To induce definitive endoderm into anterior foregut endoderm, cells were cultured in cSFMD supplemented with 10 μM SB431542 (Tocris) and 2 μM dorsomorphin (Stemgent) for 3 d. The cells were then cultured for 9 d in cSFDM containing 3 μM CHIR99021 (Tocris), 10 ng ml^−1^ recombinant human BMP4 (PeproTech) and 100 nM retinoid acid (Millipore-Sigma) to induce NKX2.1^+^ lung progenitors. The NKX2.1^+^ lung progenitors were enriched by sorting CD47^hi^CD26^−^ cells and then resuspended in growth factor reduced Matrigel (Corning) at a density of 100,000 cells ml^−1^ and pipetted (50 μl droplets) onto the base of tissue-culture plates. The three-dimensional culture was maintained in alveolar medium composed of cSFDM containing 3 μM CHIR99021, 10 ng ml^−1^ recombinant human KGF (PeproTech), 50 nM dexamethasone, 100 nM 8-bromoadenosine 3′,5′-cyclic monophosphate sodium salt (Millipore-Sigma) and 100 nM 3-isobutyl-1-methylxan-thine (IBMX; Millipore-Sigma).

### AWO differentiation

First, hPSCs were induced to NKX2.1^+^ lung progenitors as described earlier (‘ALO differentiation’ section). The NKX2.1^+^ lung progenitors were then resuspended in growth factor reduced Matrigel (Corning) at a density of 100,000 cells ml^−1^ and pipetted (50 μl droplets) onto the base of tissue-culture plates. The three-dimensional culture was maintained in alveolar medium composed of cSFDM containing 250 ng ml^−1^ FGF2 (rhFGFbasic; R&D Systems), 100 ng ml^−1^ FGF10, 50 nM dexamethasone, 100 nM 8-bromoadenosine 3′,5′-cyclic monophosphate sodium salt (Millipore-Sigma), 100 nM 3-isobutyl-1-methylxan-thine (Millipore-Sigma) and 10 μM Y-27632 (Tocris).

### Plasmid construction and lentivirus production

A lentiviral vector containing FLAG-tagged complementary DNA to the open reading frame of human *CIART*, pLV-Puro-EF1A > hCIART/FLAG, was purchased from VectorBuilder. For CRISPR-mediated gene knockout, sgRNA sequences (listed in Supplementary Table 1) were cloned into lentiCRISPRv2 vector (Addgene, cat. no. 52961) according to the instructions described by the Zhang laboratory (https://media.addgene.org/cms/filer_public/53/09/53091cde-b1ee-47ee-97cf-9b3b05d290f2/lenticrisprv2-and-lentiguide-oligo-cloning-protocol.pdf). Lentivirus was produced in 10-cm-diameter petri dishes from HEK293T cells at 70–80% confluency that had been transfected with lentiviral plasmid and the packaging plasmids pMD2.G and psPAX2 (Addgene, cat. no. 12259 and cat. no.12260).

### SARS-CoV-2 propagation and infection

SARS-CoV-2 isolate USA-WA1/2020 (NR-52281) was provided by the Center for Disease Control and Prevention and obtained through BEI Resources, NIAID, NIH. SARS-CoV-2 was propagated in Vero E6 cells (CRL-1586) in DMEM supplemented with 2% FBS, 4.5 g l^−1^ d-glucose, 4 mM l-glutamine, 10 mM non-essential amino acids, 1 mM sodium pyruvate and 10 mM HEPES using a passage-2 stock of virus as described previously^41^. Three days after infection virus-containing supernatants were purified as described previously^42^. Briefly, supernatant containing propagated virus was filtered through an Amicon Ultra 15 (100 kDa) centrifugal filter (Millipore-Sigma) at approximately 3,000*g* for 20 min. The flow through was discarded and virus was resuspended in DMEM supplemented as described earlier. The infectious titres of SARS-CoV-2 were determined by plaque assay in Vero E6 cells in Minimum Essential Media supplemented with 2% FBS, 4 mM l-glutamine, 0.2% BSA, 10 mM HEPES, 0.12% NaHCO_3_ and 0.7% agar. All m.o.i. values were based on the titre determined from plaque assays on Vero E6 cells. All work involving live SARS-CoV-2 was performed in the CDC and USDA-approved biosafety level 3 facilities of the Icahn School of Medicine at Mount Sinai and NYU Langone in accordance with institutional biosafety requirements.

### scRNA-seq sample preparation

*CIART*^−/−^ and wild-type hPSC-AWOs were infected with SARS-CoV-2 (m.o.i. = 0.1). The hPSC-AWOs were dissociated into a single-cell suspension using Accutase cell detachment solution (Innovative Cell Technologies) at 24 h.p.i. and incubated at 37 °C for approximately 5 min, followed by gentle pipetting to break apart groups of cells. The cells were then washed twice in 1×PBS and filtered using a 40-μm Flowmi cell strainer (Bel-Art Scienceware). Cell counts and viability were then determined using trypan blue staining and a Countess II automated cell counter (Thermo Fisher Scientific). Target cell inputs of 10,000 cells for each condition were then loaded into a Chromium Controller using Chromium Next GEM (Gel Bead-In Emulsion) single cell 5′ library and gel bead kit v1.1 (10x Genomics) according to the manufacturer’s instructions. After the generation of GEMs, cDNA synthesis and library preparation of all samples was completed using the Chromium single cell 5′ library kit v1.1 (10x Genomics) according to the manufacturer’s instructions.

### scRNA-seq data analysis

The 10x libraries were sequenced on an Illumina NovaSeq6000 sequencer with pair-end reads (28 base pairs (bp) for read 1 and 91 bp for read 2). The sequencing data were primarily analysed by the 10x cellranger pipeline (v5.0.0) in two steps. In the first step, cellranger mkfastq demultiplexed samples and generated fastq files, and in the second step, cellranger count aligned fastq files to the reference genome and extracted gene-expression UMI counts matrices. To measure both human and viral gene expression, we built a custom reference genome by integrating the SARS-CoV-2 virus genome into the 10x pre-built human reference (GRCh38 v3.0.0) using cellranger mkref. The SARS-CoV-2 virus genome (NC_045512.2) was downloaded from NCBI.

Cell-free messenger RNA contamination in each scRNA-seq sample was estimated and removed using the R SoupX package (v1.6.1). Specifically, the cell-free mRNA expression profiles were estimated based on empty-droplet information stored in the 10x unfiltered feature barcode matrix and the contamination fraction in each cell was estimated after incorporating the clustering information produced by the cellranger pipeline using the autoEstCont function; the UMI counts matrix was then corrected to remove the contamination using the adjustCounts function.

Putative doublet cells were discarded using the R DoubletFinder package (v2.0.3), with an expected multiple rate of 0.8% per 1,000 cells according to the 10x Genomics guideline.

We filtered cells with fewer than 500 or more than 8,000 detected genes, cells with fewer than 1,000 or more than 70,000 detected UMIs as well as cells with mitochondria gene content greater than 15%, and used the remaining cells (2,851 cells for WT + mock sample, 3,337 cells for WT + SARS-CoV-2 sample, 6,408 cells for *CIART*^−/−^ + mock sample and 4,503 cells for *CIART*^−/−^ + SARS-CoV-2 sample) for downstream analysis. We filtered doublets using DoubletFinder.

We normalized the gene-expression UMI counts using a deconvolution strategy implemented by the R scran package (v.1.14.1). In particular, we pre-clustered cells using the quickCluster function, computed size factor per cell within each cluster and rescaled the size factors by normalization between clusters using the computeSumFactors function, normalized the UMI counts per cell by the size factors and took a logarithm transform using the normalize function. We further normalized the UMI counts across samples using the multiBatchNorm function in the R batchelor package (v1.2.1).

We identified highly variable genes using the FindVariableFeatures function in the R Seurat package (v3.1.0)^43^ and selected the top 3,000 variable genes after excluding mitochondrial, ribosomal, viral and dissociation-related genes. The list of dissociation-related genes was originally built on mouse data; we converted them to human orthologue genes using Ensembl BioMart. We aligned the four samples based on their mutual nearest neighbours using the fastMNN function in the R batchelor package; this was done by performing a PCA analysis on the highly variable genes and then correcting the principal components according to their mutual nearest neighbours. We selected the corrected top 50 principal components for downstream visualization and clustering analysis.

We ran UMAP dimensional reduction using the RunUMAP function in the R Seurat package with the number of neighbouring points set to 35 and training epochs set to 2,500. We clustered cells into 17 clusters by constructing a shared nearest neighbour graph and then grouping cells of similar transcriptome profiles using the FindNeighbors and FindClusters functions (resolution set to 0.2) in the R Seurat package. We identified marker genes for each cluster by performing differential expression analysis between cells inside and outside that cluster using the FindMarkers function in the R Seurat package. We compared the marker genes of each cluster against a reference human adult lung dataset and performed an unsupervised hierarchical clustering on these 17 clusters based on their marker gene similarity to the reference. We then merged the 17 clusters into five clusters representing basal cells, ciliated-like cells 1, ciliated-like cells 2, proliferating basal cells and neuroendocrine cells based on the hierarchical clustering and UMAP results, and used them for downstream analysis. We generated UMAP plots illustrating the five clusters as well as highlighting expressions of selected genes using the R ggplot2 package.

To validate the identity of the cell populations in hPSC-AWOs, we extracted the marker genes of the five clusters in hPSC-AWOs with adjusted *P* < 0.01 and average log_2_(fold change) > 0, and compared them to the marker genes of various cell types in a human adult lung dataset^32^. We calculated the fraction of overlapping marker genes between hPSC-AWO and human adult lung cell clusters^32^.

We presented the expression difference in the SARS-CoV-2 genes between WT and *CIART*^−/−^ samples by dot plot using the DotPlot function in the R Seurat package, where the size of a dot indicates the percentage of cells that express a gene and the colour represents the relative expression level of a gene.

To evaluate viral infections of WT and *CIART*^−/−^ samples, we calculated the percentage of viral UMI counts of WT and *CIART*^−/−^ hPSC-AWOs under mock- or SARS-CoV-2-infection conditions. We considered a cell SARS-CoV-2^+^ if its viral UMI count was greater than 20 and calculated the percentage of SARS-CoV-2^+^ cells in the five cell clusters in SARS-CoV-2-infected WT and *CIART*^−/−^ samples. The virus infection results were presented as bar and violin plots using the R ggplot2 package.

### Bulk RNA-seq data analysis

The cDNA libraries were sequenced on a NovaSeq6000 sequencer with pair-end 51 bp. The sequencing reads were checked for quality using FastQC v0.10.1 and cleaned by trimming the adaptor sequences and low-quality bases using cutadapt v1.18. To measure both human and viral gene expression, the cleaned reads were aligned to the human reference genome (GRCh37) combined with the SARS-CoV-2 genome (NC_045512.2) using STAR aligner v.2.5.2b. Raw gene counts were quantified using HTSeq-count v0.11.2. We performed differential expression analysis on the gene counts using the R DESeq2 package v1.26.0, applied regularized logarithm transformation to the count data and used the transformed data for sample clustering. In particular, we performed PCA using the plotPCA function and performed an unsupervised hierarchical clustering using Euclidean distance in the R pheatmap package.

We screened for changes in gene expression across multiple hPSC-derived organoids following SARS-CoV-2 infection at various m.o.i. We studied nine conditions (three organoids at three m.o.i.) and pre-filtered candidate genes for each condition with base mean > 10, adjusted *P* value < 0.05 and log_2_(fold change) > 0.75, and searched for genes shared by at least seven conditions.

We observed high viral reads for AWOs and ALOs following SARS-CoV-2 infection, resulting in a lower count of human genes than the mock samples. To make a fair comparison, we downsampled the raw gene counts in all AWOs and ALOs so that their human gene counts were comparable.

### ATAC–seq data analysis

The ATAC-seq libraries were sequenced on a NovaSeq6000 sequencer with pair-end 51 bp. The sequencing reads were checked for quality using FastQC v0.11.9 (ref. ^44^) and trimmed to remove adaptor sequences and low-quality bases using cutadapt v3.4 (ref. ^45^). The trimmed reads were aligned to the human GRCh37 reference genome using Bowtie2 v2.4.4 (ref. ^45^) with the parameters -X 2000–very-sensitive -k 5. Duplicate reads were discarded using Picard v2.26.2. Peaks were identified for each replicate sample using Genrich v0.6.1 with the parameters -j -q 0.05 -a 200.0, -e to remove mitochondrial genome and regions not assembled into chromosomes, and -E to exclude ‘N’ homopolymers or high mappability regions in the genome. The called peaks were loaded into the R DiffBind package v3.2.1 for downstream differential binding analysis. Briefly, consensus peaks were calculated for the WT and *CIART*^−/−^ conditions by combining peaks that overlap in at least two replicate samples in each condition, and a consensus peak set was generated by taking a union of peaks from both conditions and filtering peaks in the ENCODE blacklisted regions. Reads overlapping the consensus peak set were counted for each sample and background normalization was applied to the read counts. Differential binding sites between WT and *CIART*^−/−^ conditions were identified with false detection rate (FDR) < 0.05.

To view the peaks in the ATAC-seq data, reads in the binary alignment map (BAM) files were shifted +4 bp and −5 bp for the positive and negative strand, respectively, using alignmentSieve from the deepTools package^46^ v3.5.1 to account for the 9-bp duplication created by DNA repair of the nick by Tn5 transposase^47^. Coverage tracks in bigWig format were generated based on the shifted BAM files using bamCoverage from the deepTools package v3.5.1 (ref. ^46^) and visualized using the R karyoploteR package v1.18.0 (ref. ^48^).

### CUT&RUN assay

CUT&RUN was performed using CUTANA ChIC/CUT&RUN Kit (Epicypher) according to the user manual. Briefly, differentiation day 30 hPSC-CMs were transduced with lentiviruses encoding FLAG-tagged cDNA to the open reading frame of human *CIART*. Seven days after transduction, 1 × 10^6^ cells per sample were washed and bound to 11 μl of activated Concanavalin A beads (Epicypher). The bead-bound cells were incubated with monoclonal anti-FLAG M2 (Sigma Aldrich; 1:100) at 4 °C overnight. After washing, the cells were incubated with CUTANA pAG-MNase (Epicypher) for 10 min, targeted chromatin tagmentation was initiated by the addition of 100 mM CaCl_2_ and allowed to proceed for 2 h at 4 °C, and then stop buffer containing 0.5 ng *Escherichia coli* spike-in DNA was added to each sample. Released chromatin fragments were purified using DNA Clean & Concentrator-5 (Zymo Research). Libraries were generated using a NEBNext ultra II DNA library prep kit and sequenced on an Illumina NovaSeq (PE-00) at the Weill Cornell Medical College Genomics Core.

### CUT&RUN data analysis

The CUT&RUN libraries were sequenced on a NovaSeq6000 sequencer with pair-end 51 bp. The sequencing reads were processed using the CUT&RUNTools package^49^ using the default settings. Briefly, reads were adaptor trimmed using Trimmomatic v0.36 (ref. ^50^) and an additional trimming step was performed to remove up to 6 bp adaptor from each read. Next, the reads were aligned to the hg19 genome using Bowtie2 v2.2.9 (ref. ^51^) with the ‘dovetail’ settings enabled. Alignments were further divided into ≤120-bp and >120-bp fractions. Alignments from the ≤120-bp fractions were used for peak calling with MACS2 v2.1.1 (ref. ^52^). Candidate peaks were extracted by generating a consensus peak set based on the two WT replicate samples and then subtracting peaks overlapping with the negative control sample using the R DiffBind package v3.2.7. The candidate peaks were annotated and visualized using the R ChIPseeker^53^ package v1.28.3. To identify the downstream targets regulated by CIART in hPSC-CMs, the candidate peaks were further filtered by incorporating the ATAC–seq and bulk RNA-seq data. In particular, we selected peaks for which chromatin accessibility changes were observed in promoter regions of the corresponding genes, with FDR < 0.05, and gene expression changes were observed in the corresponding genes, with adjusted *P* < 0.1 and absolute log_2_(fold change) > 1. To view the peaks in the CUT&RUN and ATAC–seq data, coverage tracks in bigWig format were generated based on the BAM files using bamCoverage from the deepTools^46^ package v3.5.1 and visualized using the R karyoploteR^48^ package v1.18.0. Although there were very small peaks at the *NR4A1*-associated region in the control CUT&RUN track, these peaks were too weak to be considered real signals.

### Immunofluorescence staining and confocal microscopy

Cells or organoids were fixed with 4% paraformaldehyde at room temperature for 30 min. The samples were then blocked and permeabilized in PBS containing 5% horse serum and 0.1% Triton X-100 for 1 h at room temperature and incubated with primary antibodies at 4 °C overnight, followed by incubation with fluorescence-conjugated secondary antibodies at room temperature for 1 h. Nuclei were counterstained with 4,6-diamidino-2-phenylindole (DAPI). Information on the antibodies used for immunofluorescence staining is provided in Supplementary Table 2. Images were taken using a Zeiss LSM 800 confocal microscope and scored using the MetaMorph image analysis software (Molecular Devices).

### Flow cytometry analysis

Intracellular staining was performed following the instructions in the user manual for the Fixation and permeabilization solution kit (BD Biosciences). Briefly, cells were dissociated and resuspended in Fixation and Permeabilization solution for 20 min at 4 °C and washed twice in 1×Perm and Wash buffer. The fixed cells were incubated with primary antibody at 4 °C overnight, washed three times with 1×Perm and Wash buffer, and then incubated with fluorescence-conjugated secondary antibody for 1 h at 4 °C in the dark. The cells were washed three times before flow cytometry analysis using an Attune NxT instrument and the data were processed using the FlowJo v10 software. Information on the antibodies used for flow cytometry is provided in Supplementary Table 2.

### Western blotting

Total protein was extracted from WT and *CIART*^−/−^ H1-hESCs, hPSC-ALOs, hPSC-AWOs and hPSC-CMs using RIPA buffer (Sigma) supplemented with protease and phosphatase inhibitor cocktail (Thermo Fisher). The protein samples were loaded onto NuPAGE 4–12% bis-Tris protein gels (Thermo Fisher), resolved by electrophoresis and transferred onto nitrocellulose membranes. The membranes were incubated with the following primary antibodies: GAPDH rabbit monoclonal antibody (Cell Signaling, cat. no. 5174S; 1:1,000) and CIART polyclonal antibody (Thermo Fisher, PA5-55643; 1:1,000). The primary antibodies were detected by fluorophore-conjugated secondary donkey anti-rabbit (IRDye 800CW, 926-32213; 1:15,000). Information on the antibodies used for western blotting is provided in Supplementary Table 2.

### qRT-PCR

Total RNA samples were prepared from cells/organoids using TRIzol and a Direct-zol RNA miniprep plus kit (Zymo Research) according to the manufacturer’s instructions. To quantify viral replication, measured by the accumulation of subgenomic *N* transcripts, one-step qRT-PCR was performed using a SuperScript III platinum SYBR Green one-step qRT-PCR kit (Invitrogen) with primers specific for the TRS-L and TRS-B sites for the *N* gene as well as *ACTB* as an internal reference, as described previously^1^. The qRT-PCR reactions were performed on an Applied Biosystems QuantStudio 6 flex real-time PCR instrument. The delta-delta-cycle threshold (DD*C*_t_) was determined relative to the *ACTB* and mock infected/treated samples. The sequences of primers/probes are provided in Supplementary Table 3.

### Metabolic profiling

Samples were extracted using 200 µl of 4:1 (vol/vol) methanol:water (containing internal standards) and quickly frozen in liquid nitrogen and thawed on ice. The thawed samples were sonicated for 2 min. The freeze–thaw–sonication procedure was repeated twice. Next, the samples were placed at −20 °C for 10 min and centrifuged at 13,523*g* for 10 min; the supernatant was transferred to a sampling vial. The samples were injected into a liquid chromatography-coupled mass spectrometry system (LC–MS; Waters UPLC coupled with ABSciex 6500+ QTrapMS) for acyl coenzyme A analysis. Following the LC–MS analysis, the samples were dried under gentle nitrogen flow, derivatized with a two-step derivatization procedure and analysed by gas chromatography–mass spectrometry for untargeted metabolomics. The derivatization was first methoximized with 50 µl methoxyamine hydrochloride (15 mg ml^−1^ in pridine) at 30 °C for 90 min. The silylation step was done with 50 µl *N*,*O*-bis(trimethylsilyl)trifluoroacetamide (containing 1% trimethylsilyl chloride) at 70 °C for 60 min. The samples were analysed by gas chromatography mass spectrometry (8890 GC with 5977B MS; Agilent). The LC–MS data were analysed using the Mutiquant (ABSciex) software and the gas chromatography–mass spectrometry data were analysed using the MS refiner (Genedata) software.

### Statistics and reproducibility

All statistical analyses were performed using the GraphPad Prism 6 software. Data are shown as the mean ± s.d. Data distribution was assumed to be normal but this was not formally tested. For two-group data, we used a two-tailed unpaired Student’s *t*-test. For one-independent-variable data, we used a one-way analysis of variance. *P* < 0.05 was considered statistically significant. No data were excluded from the analyses. No statistical method was used to pre-determine sample size but our sample sizes are similar to those reported in previous publications^2,54^. Samples were assigned randomly. All experiments were performed at least three independent times with similar results, unless specified otherwise in the figure legends. Descriptions of each statistical test and the *n* and *P* values are included in each legend or experimental Source Data. All investigators analysing the data were blinded to the sample name.

### Reporting summary

Further information on research design is available in the Nature Portfolio Reporting Summary linked to this article.

## Online content

Any methods, additional references, Nature Portfolio reporting summaries, source data, extended data, supplementary information, acknowledgements, peer review information; details of author contributions and competing interests; and statements of data and code availability are available at 10.1038/s41556-023-01095-y.

## Supplementary information

Reporting Summary

Supplementary Table Supplementary Table 1. Sequences of sgRNAs used in gene targeting.
Supplementary Table 2. Antibodies used for immunostaining, intracellular flow cytometry analysis, cut&run and western blot.
Supplementary Table 3. Sequences of primers used for qRT-PCR.

## Data Availability

The RNA-seq and scRNA-seq data that support the findings of this study have been deposited in the Gene Expression Omnibus under the accession code GSE202967 (including GSE202963, GSE202964 and GSE202965). Source data are provided with this paper. All other relevant data are available from the corresponding author on reasonable request.

## Source data

Source Data Fig. 1 Statistical source data.
Source Data Fig. 2 Statistical source data.
Source Data Fig. 3 Statistical source data.
Source Data Fig. 4 Statistical source data.
Source Data Extended Data Fig. 1 and Table 1 Flow cytometry gating strategy.
Source Data Extended Data Fig. 2 and Table 2 Statistical source data.
Source Data Extended Data Fig. 3 and Table 3 Unprocessed western blots.
Source Data Extended Data Fig. 5 and Table 5 Statistical source data.
Source Data Extended Data Fig. 7 and Table 7 Statistical source data.
Source Data Extended Data Fig. 8 and Table 8 Statistical source data.

## Code availability

The code used for data analysis has been deposited to GitHub (https://github.com/shuibingchen/COVID-19_Multi).
