# Supplementary material for: A multi-organoid platform identifies CIART as a key factor for SARS-CoV-2 infection
Source: Nat Cell Biol. 2023 Mar 13;25(3):381–9. doi: 10.1038/s41556-023-01095-y (PMC10014579; doi:10.1038/s41556-023-01095-y)
Supplement: Supplementary file 1 — Reporting Summary [file 41556_2023_1095_MOESM1_ESM.pdf]

nature portfolio

Corresponding author(s): Shuibing Chen
Todd Evans
Benjamin R. tenOever
Robert E. Schwartz

Last updated by author(s): Dec 14, 2022

Reporting Summary

Nature Portfolio wishes to improve the reproducibility of the work that we publish. This form provides structure for consistency and transparency in reporting. For further information on Nature Portfolio policies, see our Editorial Policies and the Editorial Policy Checklist.

Statistics

For all statistical analyses, confirm that the following items are present in the figure legend, table legend, main text, or Methods section.

n/a	Confirmed	
☐	☒	The exact sample size (*n*) for each experimental group/condition, given as a discrete number and unit of measurement
☐	☒	A statement on whether measurements were taken from distinct samples or whether the same sample was measured repeatedly
☐	☒	The statistical test(s) used AND whether they are one- or two-sided<br>*Only common tests should be described solely by name; describe more complex techniques in the Methods section.*
☐	☒	A description of all covariates tested
☐	☒	A description of any assumptions or corrections, such as tests of normality and adjustment for multiple comparisons
☐	☒	A full description of the statistical parameters including central tendency (e.g. means) or other basic estimates (e.g. regression coefficient) AND variation (e.g. standard deviation) or associated estimates of uncertainty (e.g. confidence intervals)
☐	☒	For null hypothesis testing, the test statistic (e.g. *F*, *t*, *r*) with confidence intervals, effect sizes, degrees of freedom and *P* value noted<br>*Give P values as exact values whenever suitable.*
☒	☐	For Bayesian analysis, information on the choice of priors and Markov chain Monte Carlo settings
☒	☐	For hierarchical and complex designs, identification of the appropriate level for tests and full reporting of outcomes
☒	☐	Estimates of effect sizes (e.g. Cohen's *d*, Pearson's *r*), indicating how they were calculated

Our web collection on statistics for biologists contains articles on many of the points above.

Software and code

Policy information about availability of computer code

Data collection	Applied Biosystems QuantStudio 6 Flex Real-Time PCR, Zeiss LSM 800 confocal microscope, 10X Chromium system, Attune NxT, NovaSeq6000 sequencer
Data analysis	Graphpad Prism 6, MetaMorph image analysis software, Flowjo v10 software, 10X cellranger, R Seurat package, R SoupX package v1.6.1, R DoubletFinder package v2.0.3, R scran package v.1.14.1, R batchelor package v1.2.1, R batchelor package, R ggplot2 package, STAR aligner v.2.5.2b, R DESeq2 package v1.26.0, R pheatmap package, FastQC v0.11.9, cutadapt v3.4, bowtie2 v2.4.4, picard v2.26.2, Genrich v0.6.1, R DiffBind package v3.2.1, deepTools package v3.5.1, R karyoploteR package v1.18.0, CUT&RUNTools package, Trimmomatic v0.36, bowtie2 v2.2.9, MACS2 v2.1.1, R DiffBind package v3.2.7, R ChIPseeker package v1.28.3

For manuscripts utilizing custom algorithms or software that are central to the research but not yet described in published literature, software must be made available to editors and reviewers. We strongly encourage code deposition in a community repository (e.g. GitHub). See the Nature Portfolio guidelines for submitting code & software for further information.

Data

Policy information about availability of data

All manuscripts must include a data availability statement. This statement should provide the following information, where applicable:
- Accession codes, unique identifiers, or web links for publicly available datasets
- A description of any restrictions on data availability
- For clinical datasets or third party data, please ensure that the statement adheres to our policy

RNA-seq and scRNA-seq data associated with this study have been deposited in the Gene Expression Omnibus (GEO) under accession code GSE202967 (including GSE202963, GSE202964, GSE202965).

Field-specific reporting

Please select the one below that is the best fit for your research. If you are not sure, read the appropriate sections before making your selection.

☒ Life sciences ☐ Behavioural & social sciences ☐ Ecological, evolutionary & environmental sciences

For a reference copy of the document with all sections, see nature.com/documents/nr-reporting-summary-flat.pdf

Life sciences study design

All studies must disclose on these points even when the disclosure is negative.

Sample size	Sample size was determined by previous studies. (Nature 589 (7841), 270-275; Cell metabolism 33 (8), 1577-1591; Cell stem cell 27 (1), 125-136)
Data exclusions	No exclusions
Replication	All attempts at replication are successful and have been included in the studies.
Randomization	Cells/organoids were randomly separated into different groups for DMSO vehicle or RXR inhibitors treatment.
Blinding	The investigators who performed SARS-CoV-2 infection were blinded to the group allocation. The investigators who prepared cells/organoids for SARS-CoV-2 infection were not blinded to group allocation because it was impossible.

Reporting for specific materials, systems and methods

We require information from authors about some types of materials, experimental systems and methods used in many studies. Here, indicate whether each material, system or method listed is relevant to your study. If you are not sure if a list item applies to your research, read the appropriate section before selecting a response.

Materials & experimental systems

n/a	Involved in the study	
☐	☒	Antibodies
☐	☒	Eukaryotic cell lines
☒	☐	Palaeontology and archaeology
☒	☐	Animals and other organisms
☒	☐	Human research participants
☒	☐	Clinical data
☒	☐	Dual use research of concern

Methods

n/a	Involved in the study	
☒	☐	ChIP-seq
☐	☒	Flow cytometry
☒	☐	MRI-based neuroimaging

Antibodies

Antibodies used

Immunostaining SARS-CoV-2 Nucleocapsid Antibody 40143-R001 #R001 Rabbit Sino Biological 1:1,000
Immunostaining SARS-CoV-2 Nucleocapsid Antibody 40143-MM08 #MM08 Mouse Sino Biological 1:1,000
Immunostaining Anti-Cardiac Troponin T antibody ab8295 #1C11 Mouse ABCAM 1:1,000
Immunostaining Anti-Sarcomeric Alpha Actinin antibody ab137346 Polyclonal Rabbit ABCAM 1:500
Immunostaining Anti-Mucin 5AC antibody ab3649 #45M1 Mouse ABCAM 1:500
Immunostaining Anti-P63 CM163A #4A4 Mouse Biocare Medical 1:500
Immunostaining FOXJ1 Monoclonal Antibody 14-9965-82 #2A5 Mouse Thermo Fisher Scientific 1:500
Immunostaining Anti-Pro-SP-B WRAB-55522 Polyclonal Rabbit Seven Hills Bioreagents 1:500
Immunostaining Anti-Mature SP-B WRAB-48604 Polyclonal Rabbit Seven Hills Bioreagents 1:500
Immunostaining Anti-Pro-SP-C WRAB-9337 Polyclonal Rabbit Seven Hills Bioreagents 1:500
Immunostaining Anti-Mature-SP-C WRAB-76694 Polyclonal Rabbit Seven Hills Bioreagents 1:500
Immunostaining Human/Mouse/Rat/Hamster ACE-2 Antibody AF933 Polyclonal Goat R&D Systems 1:500
Immunostaining Oct-4A (C30A3) Rabbit mAb 2840 #C30A3 Rabbit Cell Signaling Technologies 1:500
Immunostaining Sox2 (D6D9) Rabbit mAb 3579 #D6D9 Rabbit Cell Signaling Technologies 1:500
Immunostaining Nanog (D73G4) XP® Rabbit mAb 4903 #D73G4 Rabbit Cell Signaling Technologies 1:500
Immunostaining SSEA4 (MC813) Mouse mAb 4755 #MC813 Mouse Cell Signaling Technologies 1:500
Immunostaining TRA-1-60(S) Mouse mAb 4746 #TRA-1-60(S) Mouse Cell Signaling Technologies 1:500
Immunostaining TRA-1-81 Mouse mAb 4745 #TRA-1-81 Mouse Cell Signaling Technologies 1:500
Immunostaining Donkey anti-Mouse IgG (H+L) Highly Cross-Adsorbed Secondary Antibody, Alexa Fluor 488 A-21202 Polyclonal Donkey Thermo Fisher Scientific 1:1,000
Immunostaining Donkey anti-Mouse IgG (H+L) Highly Cross-Adsorbed Secondary Antibody, Alexa Fluor 594 A-21203 Polyclonal Donkey Thermo Fisher Scientific 1:1,000
Immunostaining Donkey anti-Rabbit IgG (H+L) Highly Cross-Adsorbed Secondary Antibody, Alexa Fluor™ Plus 488 A32790 Polyclonal

Donkey Thermo Fisher Scientific 1:1,000
Immunostaining Donkey anti-Rabbit IgG (H+L) Secondary Antibody, Alexa Fluor 594 conjugate A-21207 Polyclonal Donkey Thermo Fisher Scientific 1:1,000
Immunostaining Donkey anti-Goat IgG (H+L) Cross-Adsorbed Secondary Antibody, Alexa Fluor 488 A-11055 Polyclonal Donkey Thermo Fisher Scientific 1:1,000
FACS FITC Mouse Anti-Human CD47 556045 #B6H12 Mouse BD Biosciences 1:200
FACS APC Mouse Anti-Human CD26 563670 #M-A261 Mouse BD Biosciences 1:200
Flow cytometry Anti-Cardiac Troponin T antibody ab8295 #1C11 Mouse ABCAM 1:1,000
Flow cytometry Anti-Sarcomeric Alpha Actinin antibody ab137346 Polyclonal Rabbit ABCAM 1:500
Flow cytometry Donkey anti-Mouse IgG (H+L) Highly Cross-Adsorbed Secondary Antibody, Alexa Fluor 488 A-21202 Polyclonal Donkey Thermo Fisher Scientific 1:1,000
Flow cytometry Donkey anti-Rabbit IgG (H+L) Secondary Antibody, Alexa Fluor 647 conjugate A-31573 Polyclonal Donkey Thermo Fisher Scientific 1:1,000
CUT&RUN Anti-FLAG® M2 antibody F1804 #M2 Mouse Sigma Aldrich 1:100
CUT&RUN Recombinant mouse IgG1 monoclonal isotype control ab280974 #R312-MouseIgG1 Mouse ABCAM 1:100
Western Blot GAPDH (D16H11) XP® Rabbit mAb 5174S #D16H11 Rabbit Cell Signaling Technologies 1:1,000
Western Blot CIART Antibody NBP1-88685 Polyclonal Rabbit NOVUS Biologicals 1:500
Western Blot IRDye® 800CW Donkey anti-Rabbit IgG Secondary Antibody 926-32213 Polyclonal Donkey LI-COR 1:15,000

Validation

The antibodies were validated by either western blotting or immunostaining by vendors or previously published paper. please refer to the vendor website or previously published paper as listed after each antibody below for the detailed validation conditions and results.
SARS-CoV-2 Nucleocapsid Antibody Rabbit 40143-R001 Sino Biological https://www.sinobiological.com/antibodies/cov-nucleocapsid-40143-r001
SARS-CoV-2 Nucleocapsid Antibody Mouse 40143-MM08 Sino Biological https://www.sinobiological.com/antibodies/cov-nucleocapsid-40143-mm08
Anti-Cardiac Troponin T antibody Mouse ab8295 ABCAM https://www.abcam.com/cardiac-troponin-t-antibody-1c11-ab8295.html
Anti-Sarcomeric Alpha Actinin antibody Rabbit ab137346 ABCAM https://www.abcam.com/sarcomeric-alpha-actinin-antibody-ab137346.html
Anti-Mucin 5AC antibody Mouse ab3649 ABCAM https://www.abcam.com/mucin-5ac-antibody-45m1-ab3649.html
Anti-P63 Mouse CM163A Biocare Medical https://biocare.net/product/p63-antibody/
FOXJ1 Monoclonal Antibody Mouse 14-9965-82 Thermo Fisher Scientific https://www.thermofisher.com/antibody/product/FOXJ1-Antibody-clone-2A5-Monoclonal/14-9965-95
Anti-Pro-SP-B Rabbit WRAB-55522 Seven Hills Bioreagents https://www.sevenhillsbioreagents.com/products/anti-pro-sp-b-rabbit
Anti-Mature SP-B Rabbit WRAB-48604 Seven Hills Bioreagents https://www.nature.com/articles/s41586-020-2901-9#Sec34
Anti-Pro-SP-C Rabbit WRAB-9337 Seven Hills Bioreagents https://www.sevenhillsbioreagents.com/products/anti-pro-sp-c-rabbit-n-terminal
Anti-Mature-SP-C Rabbit WRAB-76694 Seven Hills Bioreagents https://www.sevenhillsbioreagents.com/products/anti-mature-sp-c-rabbit
Human/Mouse/Rat/Hamster ACE-2 Antibody Goat AF933 R&D Systems https://www.rndsystems.com/products/human-mouse-rat-hamster-ace-2-antibody_af933
Oct-4A (C30A3) Rabbit mAb Rabbit 2840 Cell Signaling Technologies https://www.cellsignal.com/products/primary-antibodies/oct-4a-c30a3-rabbit-mab/2840
Sox2 (D6D9) Rabbit mAb Rabbit 3579 Cell Signaling Technologies https://www.cellsignal.com/products/primary-antibodies/sox2-d6d9-xp-rabbit-mab/3579
Nanog (D73G4) XP® Rabbit mAb Rabbit 4903 Cell Signaling Technologies https://www.cellsignal.com/products/primary-antibodies/nanog-d73g4-xp-rabbit-mab/4903
SSEA4 (MC813) Mouse mAb Mouse 4755 Cell Signaling Technologies https://www.cellsignal.com/products/primary-antibodies/ssea4-mc813-mouse-mab/4755
TRA-1-60(S) Mouse mAb Mouse 4746 Cell Signaling Technologies https://www.cellsignal.com/products/primary-antibodies/tra-1-60-s-tra-1-60-s-mouse-mab/4746
TRA-1-81 Mouse mAb Mouse 4745 Cell Signaling Technologies https://www.cellsignal.com/products/primary-antibodies/tra-1-81-tra-1-81-mouse-mab/4745
Donkey anti-Mouse IgG (H+L) Highly Cross-Adsorbed Secondary Antibody, Alexa Fluor 488 Donkey A-21202 Thermo Fisher Scientific https://www.thermofisher.com/antibody/product/Donkey-anti-Mouse-IgG-H-L-Highly-Cross-Adsorbed-Secondary-Antibody-Polyclonal/A-21202
Donkey anti-Mouse IgG (H+L) Highly Cross-Adsorbed Secondary Antibody, Alexa Fluor 594 Donkey A-21203 Thermo Fisher Scientific https://www.thermofisher.com/antibody/product/Donkey-anti-Mouse-IgG-H-L-Highly-Cross-Adsorbed-Secondary-Antibody-Polyclonal/A-21203
Donkey anti-Rabbit IgG (H+L) Highly Cross-Adsorbed Secondary Antibody, Alexa Fluor™ Plus 488 Donkey A32790 Thermo Fisher Scientific https://www.thermofisher.com/antibody/product/Donkey-anti-Rabbit-IgG-H-L-Highly-Cross-Adsorbed-Secondary-Antibody-Polyclonal/A32790
Donkey anti-Rabbit IgG (H+L) Secondary Antibody, Alexa Fluor 594 conjugate Donkey A-21207 Thermo Fisher Scientific https://www.thermofisher.com/antibody/product/Donkey-anti-Rabbit-IgG-H-L-Highly-Cross-Adsorbed-Secondary-Antibody-Polyclonal/A-21207
Donkey anti-Goat IgG (H+L) Cross-Adsorbed Secondary Antibody, Alexa Fluor 488 Donkey A-11055 Thermo Fisher Scientific https://www.thermofisher.com/antibody/product/Donkey-anti-Goat-IgG-H-L-Cross-Adsorbed-Secondary-Antibody-Polyclonal/A-11055
FITC Mouse Anti-Human CD47 Mouse 556045 BD Biosciences https://www.bdbiosciences.com/en-us/products/reagents/flow-cytometry-reagents/research-reagents/single-color-antibodies-ruo/fitc-mouse-anti-human-cd47.556045
APC Mouse Anti-Human CD26 Mouse 563670 BD Biosciences https://www.bdbiosciences.com/en-us/products/reagents/flow-cytometry-reagents/research-reagents/single-color-antibodies-ruo/apc-mouse-anti-human-cd26.563670
Anti-Sarcomeric Alpha Actinin antibody Rabbit ab137346 ABCAM https://www.abcam.com/sarcomeric-alpha-actinin-antibody-ab137346.html
Donkey anti-Rabbit IgG (H+L) Secondary Antibody, Alexa Fluor 647 conjugate Donkey A-31573 Thermo Fisher Scientific https://www.thermofisher.com/antibody/product/Donkey-anti-Rabbit-IgG-H-L-Highly-Cross-Adsorbed-Secondary-Antibody-Polyclonal/

A-31573
Anti-FLAG® M2 antibody Mouse F1804 Sigma Aldrich https://www.sigmaaldrich.com/US/en/product/sigma/f1804
Recombinant mouse IgG1 monoclonal isotype control Mouse ab280974 ABCAM https://www.abcam.com/mouse-igg1-monoclonal-r312-mouseigg1-isotype-control-ab280974.html
GAPDH (D16H11) XP® Rabbit mAb Rabbit 5174S Cell Signaling Technologies https://www.cellsignal.com/products/primary-antibodies/gapdh-d16h11-xp-rabbit-mab/5174
CIART Antibody Rabbit NBP1-88685 NOVUS Biologicals https://www.novusbio.com/products/ciart-antibody_nbp1-88685
IRDye® 800CW Donkey anti-Rabbit IgG Secondary Antibody Donkey 926-32213 LI-COR https://www.licor.com/bio/reagents/irdye-800cw-donkey-anti-rabbit-igg-secondary-antibody

Eukaryotic cell lines

Policy information about cell lines

Cell line source(s)	hESC line H1 (WiCell, WA01); Vero E6 (ATCC #CRL-1586); 293T (ATCC #CRL3216)
Authentication	H1 cells were authenticated by morphology and immunostaining, Vero E6 cells and 293T cells were authenticated by morphology
Mycoplasma contamination	All cell lines tested negative for mycoplasma contamination
Commonly misidentified lines (See ICLAC register)	No

Flow Cytometry

Plots

Confirm that:

☒ The axis labels state the marker and fluorochrome used (e.g. CD4-FITC).

☒ The axis scales are clearly visible. Include numbers along axes only for bottom left plot of group (a 'group' is an analysis of identical markers).

☒ All plots are contour plots with outliers or pseudocolor plots.

☒ A numerical value for number of cells or percentage (with statistics) is provided.

Methodology

Sample preparation	Flow cytometry intracellular staining was performed following the instruction of user manual of Fixation and Permeabilization Solution Kit (BD Biosciences). Briefly, cells were dissociated and resuspended in Fixation and Permeabilization solution for 20 minutes at 4°C, then washed twice in 1x Perm and Wash buffer. Fixed cells were incubated with primary antibody at 4°C overnight, washed three times with 1x Perm and Wash buffer and then incubated with fluorescence-conjugated secondary antibody for 1 hour at 4°C in the dark. Cells were washed three times prior to flow cytometry analysis.
Instrument	Accuri C6 Flow Cytometer
Software	Flowjo v10 software
Cell population abundance	Live cell populations range from 50%~75%, cTnT and a-actinin double cells account for ~75% of the live cell population.
Gating strategy	Live cell population was determined by FSC/SSC. Positive stained cells were defined by using non-stained cells as reference. Isotype control was used to confirm the specificity of staining.

☒ Tick this box to confirm that a figure exemplifying the gating strategy is provided in the Supplementary Information.
