# Supplementary figures and images for: A multi-organoid platform identifies CIART as a key factor for SARS-CoV-2 infection
Source: Nat Cell Biol. 2023 Mar 13;25(3):381–9. doi: 10.1038/s41556-023-01095-y (PMC10014579; doi:10.1038/s41556-023-01095-y)

Extended Data Fig. 1g

No staining

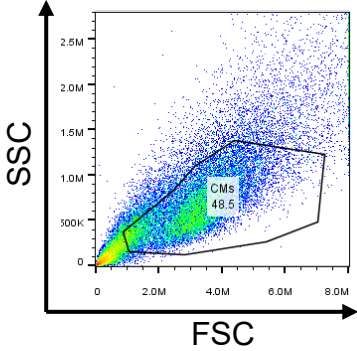

cTnT-488

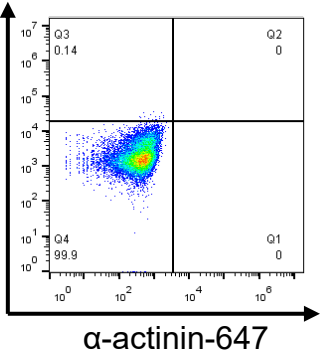

Isotype

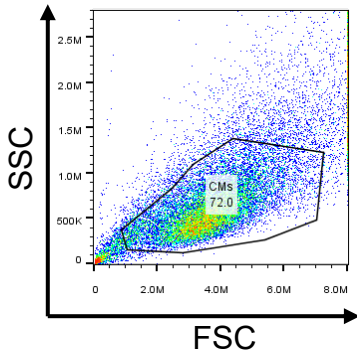

cTnT-488

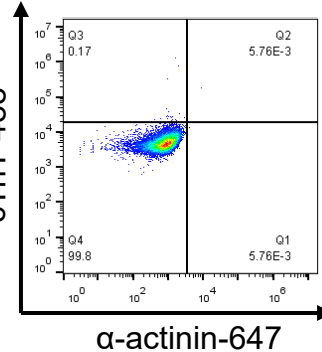

CMs

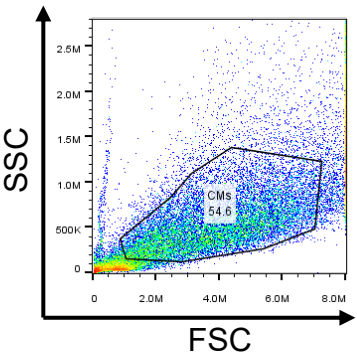

cTnT-488

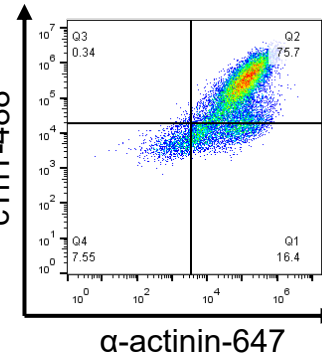

Supplement: Source Data Extended Data Fig. 1 and Table 1 — Flow cytometry gating strategy. [file 41556_2023_1095_MOESM7_ESM.pdf]

Extended Data Fig. 3d

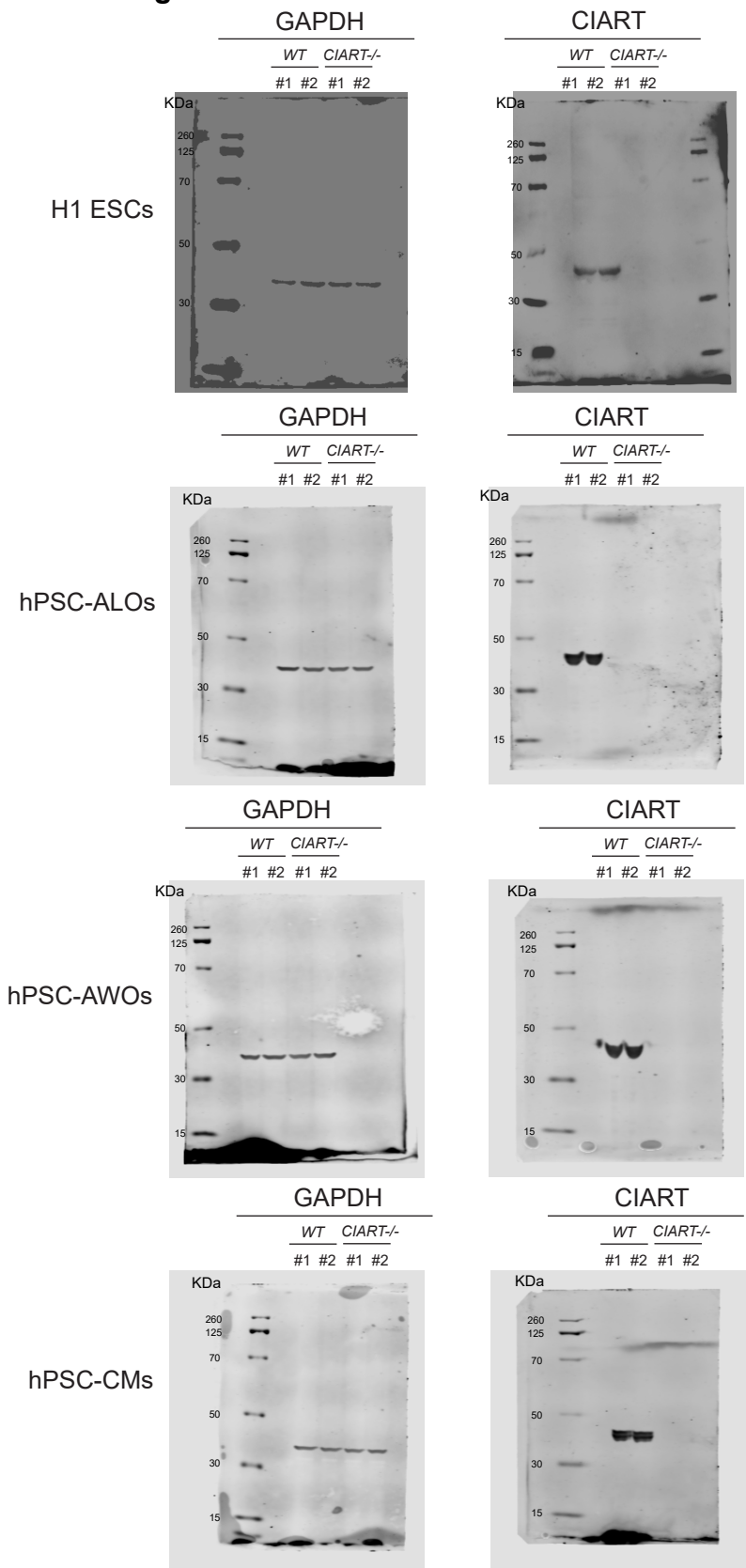

Supplement: Source Data Extended Data Fig. 3 and Table 3 — Unprocessed western blots. [file 41556_2023_1095_MOESM9_ESM.pdf]
